# Oxytocin Enhances Demethylation Through TET Enzyme Expression in Neurons of Aged Mice: Oxytocin as a Potential Antiaging Peptide

**DOI:** 10.1111/acel.70198

**Published:** 2025-08-11

**Authors:** Yuko Maejima, Shoko Yokota, Megumi Yamachi, Shizu Hidema, Tomoyuki Ono, Shu Taira, Katsuhiko Nishimori, Heidi de Wet, Kenju Shimomura

**Affiliations:** ^1^ Department of Bioregulation and Pharmacological Medicine Fukushima Medical University School of Medicine Fukushima Japan; ^2^ Departments of Obesity and Inflammation Research Fukushima Medical University School of Medicine Fukushima Japan; ^3^ Department of Physiology, Anatomy and Genetics, Sherrington Building University of Oxford Oxford UK; ^4^ Faculty of Food and Agricultural Sciences Fukushima University Fukushima Japan

**Keywords:** antiaging, methylation, mitochondria, neurons, oxytocin, TET enzyme

## Abstract

While it is well‐documented that plasma oxytocin (OXT) levels decline with age, the underlying mechanisms remain elusive. This study aimed to elucidate the physiological mechanisms contributing to this age‐related decrease in plasma OXT and the possible use of OXT supplementation on improving age‐related decline of neural function. Comparing young (9 weeks) and aged (> 45 weeks) mice, aged mice showed reduced plasma OXT levels, an increase in the inflammation marker hs‐CRP, and decreased OXT‐positive neurons in the hypothalamus. Aged mice showed signs of epigenetic changes in the hypothalamus as indicated by decreased ten‐eleven translocation (TET) family mRNA expression, decreased 5‐hydroxymethylcytosine (5hmC) positive neurons, and downregulated mitochondrial respiratory complex IV (COX IV) expression. Nasal application of OXT (10 μg/day) for 10 days to aged mice resulted in normalized plasma OXT and inflammation levels and a recovery of OXT‐positive neurons, TET2 mRNA levels, 5hmC positive neurons, and COX IV expression. Directly confirming a role for OXTR signaling, TET2, COX IV, and 5hmC in the hypothalamus and hippocampus were also found to be decreased in oxytocin receptor (OXTR) null mice, compared with age‐matched WT mice. Furthermore, we show that methylation as a result of aging decreases OXT production in hypothalamic neurons, thereby reducing circulating plasma OXT levels, which can be reversed by nasal OXT treatment. The data presented here suggest that aging, DNA methylation, mitochondrial dysfunction, inflammation, and senescence are interconnected in a vicious cycle, which can be successfully interrupted by OXT treatment.

## Graphical abstract

DNA methylation, mitochondrial dysfunction, inflammation, and senescence interact to form a vicious cycle. Here we report that oxytocin interrupts this vicious cycle by increasing TET2 expression, inducing DNA demethylation in aged neurons and raising plasma oxytocin levels, decreasing systemic inflammation.

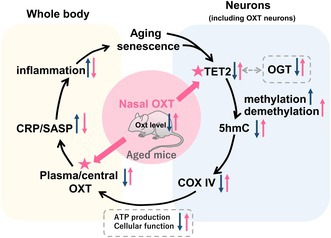

## 1 Introduction

Aging results from the accumulation of a wide variety of molecular and cellular damage over time, leading to a gradual decrease in physical and mental capacity, a growing risk of disease, and ultimately death (World Health Organization 2025). The hallmarks of aging are subdivided into three categories: molecular, cellular, and systemic level (Guo et al. 2022). Hallmarks for molecular level aging are genomic instability, telomere dysfunction, loss of proteostasis, compromised autophagy, mitochondrial dysfunction, and epigenetic alterations (Guo et al. 2022). Especially, methylation, an epigenomic alteration, plays a significant role in the aging process. Changes in DNA methylation patterns, particularly at conserved CpG sites, eventually lead to an “aged methylome”, affecting various physiological processes and potentially lifespan (Mozhui et al. 2023). Methylation has a strong impact on aging (Mozhui et al. 2023), and abnormal methylation patterns are linked to age‐related diseases, such as cancer. Therefore, the level and pattern of methylated DNA can serve as a biomarker for aging (Noroozi et al. 2021).

DNA methylation is regulated by ten‐eleven translocation (TET) enzymes (Zhang et al. 2023). TET enzymes catalyze the conversion of 5‐methylcytosine (5mC) to 5‐hydroxymethylcytosine (5hmC), 5‐formylcytosine (5fC), and finally to 5‐carboxylcytosine (5caC) (Zhang et al. 2023). DNA methylation and demethylation play an important role in numerous biological processes (Zhang et al. 2023). A decline in TET activities is frequently observed in diseases such as hematological malignancies, solid tumors (Guarnera and Jha 2024), immune dysregulation, and inflammation (Tsiouplis et al. 2020). Also, demethylation driven by TET enzymes is essential for brain development by guiding axon elongation (Singh et al. 2024). At the molecular level, 5hmC reduction as a result of knocking down TET2 induces dysfunction of mitochondria and apoptosis in hippocampal neurons (Liu et al. 2020). Because compromised autophagy and mitochondrial dysfunction are hallmarks of molecular level aging, TET is considered to be one of the major components that regulate molecular level aging.

TET2 directly interacts with O‐GlcNAc transferase (OGT), enhancing its activity in the O‐GlcNAcylation of histone H2B at transcription start sites (Chen et al. 2013). The colocalization of TET2 and OGT at transcription start sites suggests a coordinated role in gene activation (Deplus et al. 2013). Furthermore, neural O‐GlcNAcylation has been shown to improve cognitive function in the aged murine hippocampus (Wheatley et al. 2019).

It has also been reported that the TET family influences mitochondrial biogenesis by regulating genes involved in oxidative phosphorylation and mitochondrial quality control (Leon Kropf et al. 2024). COX IV, a subunit of cytochrome c oxidase (complex IV) that resides in the inner mitochondrial membrane, is functionally essential for coupling ATP production to energy requirements. Decreased COX IV levels compromise physiological membrane potential, ATP production, and overall respiratory function of mitochondria and is commonly used as a reporter of overall mitochondrial health (Arnold 2012; Li et al. 2006).

The neuropeptide oxytocin (OXT) is a hormone secreted from hypothalamic OXT neurons in the paraventricular nucleus (PVN) and supraoptic nucleus (SON) and is well‐known for its function in parturition and lactation (Dale 1906). Recent research has shown OXT to be a pleiotropic hormone with wide implications for human health far beyond its reproductive functions (Carter et al. 2020), such as maternal behavior, social behavior including pair bonding, and the perception of stress/anxiety (Jurek and Neumann 2018) and also its metabolic impact on antiobesity effects (Maejima et al. 2017).

OXT is related to aging, with plasma levels declining with age, and replenishment of OXT reverses age‐related changes in animal models (Elabd et al. 2014). As early as 1966, Bodanszky and Engel reported that OXT treatment prolongs the lifespan of rats (Bodanszky and Engel 1966). In addition, a rodent menopause model with ovariectomy (OVX) shows significant decreases in OXT levels with the development of osteoporosis. However, subcutaneous OXT treatment reverses bone loss in OVX mice (Elabd et al. 2008). Also, OXT activates autophagy and promotes hepatic regeneration (Luo et al. 2021). Therefore, optimal plasma OXT levels can be crucial for antiaging and longevity. Here, we provide novel insights into the mechanisms underlying age‐related OXT decline and highlight the potential of targeting the OXT‐TET‐DNA demethylation pathway for the development of antiaging therapies.

## 2 Results

### 2.1 Decreased Plasma OXT Level and Expression of Secretory Machinery Components of Hypothalamic OXT Neurons in Aged Animals

**FIGURE 1:**
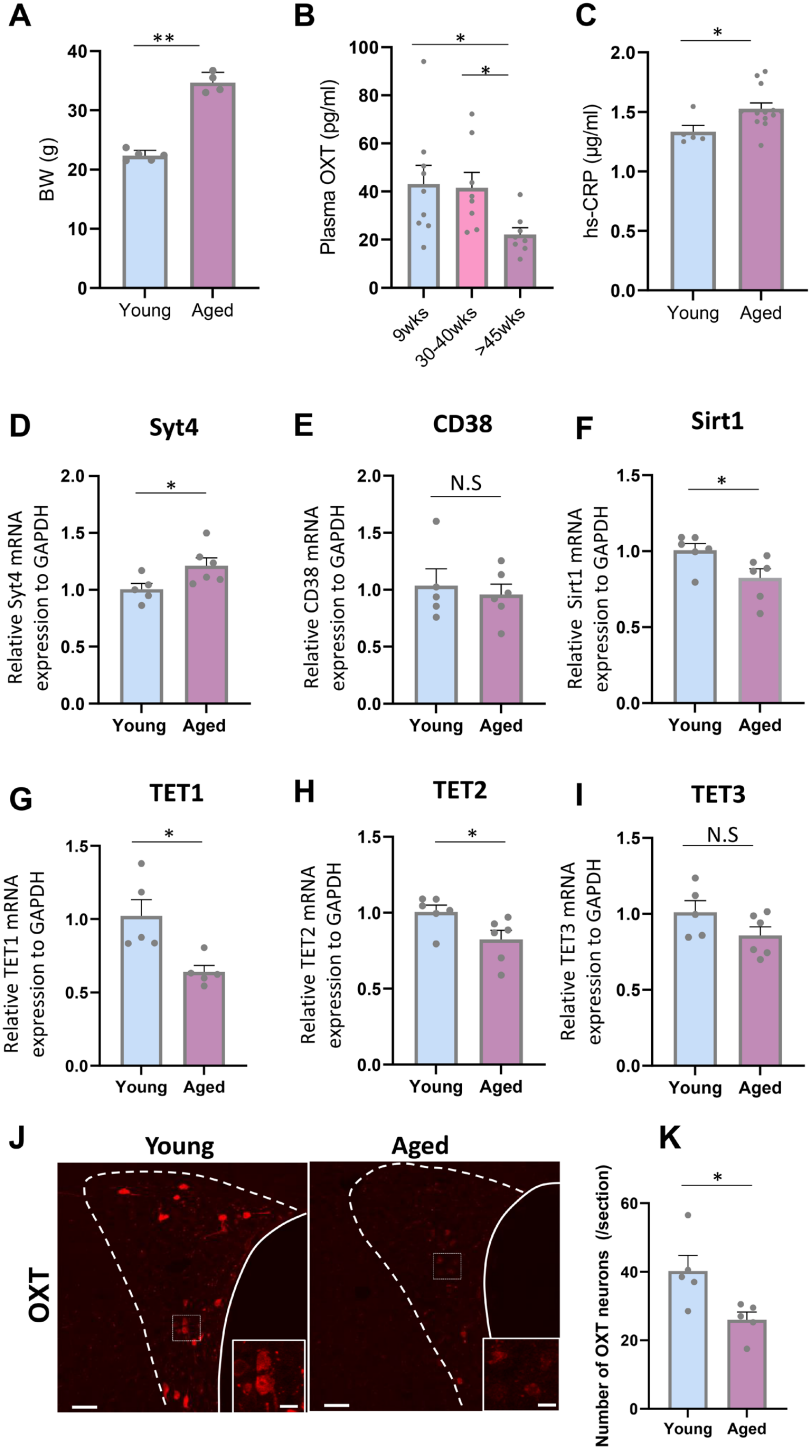
Impact of aging on the plasma OXT levels and PVN OXT expression. (A) Body weight (BW) in young (9 weeks) and aged (46–47 weeks) mice. **p < 0.01, unpaired *t*‐test, *n* = 5, 4. (B) Plasma OXT concentration in young (9 weeks), middle (34–37 weeks), and aged (47–62 weeks) mice. *p < 0.05, one‐way ANOVA followed by Holm's multiple comparison test, *n* = 9, 8, 8. (C) Plasma hs‐CRP concentration in young (9–11 weeks) and aged (47–52 weeks) mice. *p < 0.05, unpaired *t*‐test, *n* = 5, 13. (D–I) Syt4 (D), CD38 (E), Sirt1 (F), TET1 (G), TET2 (H), and TET3 (I) mRNA expression in hypothalamus of young (9 weeks) and aged (46–47 weeks) mice. *p < 0.05, ns; no significant differences, unpaired *t*‐test, *n* = 5–6. (J) Representative images of OXT immunostaining in hypothalamic paraventricular nucleus (PVN) of young (9 weeks) and aged (47 weeks) mice. Scales = 50 μm. The images located in the right bottom are enlarged images in the dotted square. Scales = 10 μm. (K) The number of OXT‐positive neurons (per section) in PVN of young and aged mice. **p < 0.01, unpaired *t*‐test, *n* = 5, 5.

Consistent with higher body weights (Figure 1A), plasma OXT levels in aged mice (> 45 weeks) were lower compared with younger mice (9 weeks) (Figure 1B). This observation would be in agreement with recent work showing decreased OXT mRNA levels in the hypothalamus of aged mice (Usmani et al. 2024), which would result in lower circulating plasma levels of OXT. Furthermore, aged mice showed significantly higher levels of the inflammation marker, high‐sensitive (hs)‐CRP (1.53 ± 0.05 μg/mL) compared with young mice (1.33 ± 0.05 μg/mL) (Figure 1C).

In order to delve into the cellular mechanisms underpinning possible OXT secretory dysfunction associated with aging, we conducted a comprehensive analysis on Synaptotagmin‐4 (Syt4) and transmembrane receptor CD38, which are essential molecular machinery governing the process of exocytosis in hypothalamic OXT neurons (Jin et al. 2007; Zhang et al. 2011). Although Syt4 mRNA expression was significantly increased in aged mice (Figure 1D), CD38 mRNA expression was not changed (Figure 1E). Also, the intensity and the number of OXT immunoreactive neurons in the paraventricular nucleus (PVN) were significantly decreased in aged mice (Figure 1J,K).

mRNA expression in the hypothalamus of the histone deacetylase sirtuin1 (Sirt1), as well as the demethylation enzymes TET1 and TET2, was significantly decreased in aged mice, while TET3 showed a tendency to decrease with age (Figure 1F–I), indicating the age‐related epigenetic changes in hypothalamic neurons.

The data presented here suggest that an age‐related decline in plasma OXT levels is correlated with decreased numbers of OXT‐positive neurons in the hypothalamus, which are subjected to age‐related epigenetic changes.

### 2.2 Nasal Treatment of OXT on Aged Mice Ameliorates Age‐Associated Changes

**FIGURE 2:**
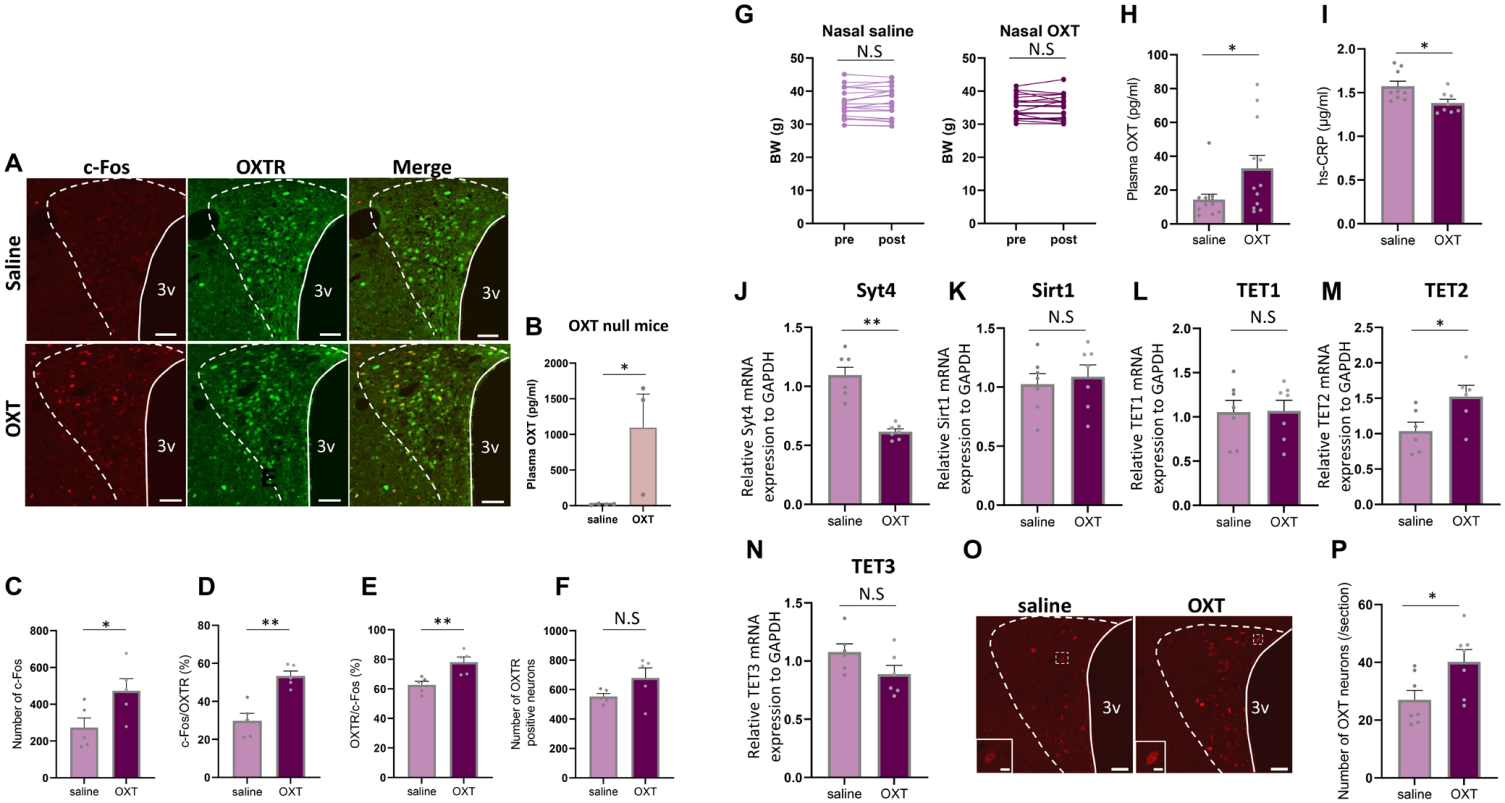
Effect of acute and subchronic nasal treatment of OXT on aged mice. (A) Representative images of c‐Fos, OXTR, and merged images at 2 h after nasal treatment of saline (upper panels) and OXT (bottom panels) to the OXTR‐Venus mice (47 weeks). Scales = 50 μm. (B) Plasma OXT concentrations in OXT null mice (12–16 weeks) 15 min after nasal treatment with saline or OXT (10 μg/10 μL). *p < 0.05; unpaired *t*‐test, *n* = 4, 3. (C) The total number of c‐Fos in the PVN of aged mice treated with saline or OXT. *p < 0.05; unpaired *t*‐test, *n* = 5, 5. (D) The percentage of c‐Fos immunoreactive neurons in OXTR positive neurons. **p < 0.01; unpaired *t*‐test, *n* = 5, 5. (E) The percentage of OXTR positive neurons in c‐Fos immunoreactive neurons. **p < 0.01; unpaired *t*‐test, *n* = 5, 5. (F) The total number of OXTR positive neurons in PVN after saline or OXT treatment. ns; no significant differences; unpaired *t*‐test, *n* = 5, 5. (G) The comparison of BW in pre‐ and posttreatment of saline (left panel) and OXT (right panel) treated aged mice (47–75 weeks, both groups were age‐matched). ns; no significant differences; unpaired *t*‐test, *n* = 23, 22. (H) Plasma OXT concentrations after saline or OXT treatment for 10 days in aged mice (47–60 weeks). *p < 0.05; unpaired *t*‐test, *n* = 12, 12. (I) Plasma CRP concentrations after saline or OXT treatment for 10 days in aged mice (47–60 weeks). *p < 0.05; unpaired *t*‐test, *n* = 9, 8. (J–N) Syt4 (J), Sirt1 (K), TET1 (L), TET2 (M), and TET3 (N) mRNA expression in the hypothalamus of aged (51 weeks) mice treated with saline or OXT. *p < 0.05, **p < 0.01, ns; no significant differences, unpaired *t*‐test, *n* = 6–7. (O) Representative images of OXT immunostaining in the hypothalamic paraventricular nucleus (PVN) of aged mice (47 weeks) treated with saline (left panel) or OXT (right panel). Scale = 50 μm. The images located in the left bottom are enlarged images in the dotted square. Scale = 10 μm. (P) The number of OXT‐positive neurons (per section) in the PVN of aged mice treated with saline or OXT. *p < 0.05, unpaired *t*‐test, *n* = 7, 7.

To examine the acute effect of nasal treatment on OXTR in the PVN of aged mice 2 h posttreatment, double immunostaining of OXTR, and neural activation marker c‐Fos was performed in aged OXTR‐Venus mice (Figure 2A). Nasal treatment of OXT (10 μg/10 μL) significantly increased plasma OXT levels in OXT null mice 15 min posttreatment (Figure 2B), indicating that the nasally administered exogenous OXT is effectively absorbed into the circulation.

As shown in Figure 2A, acute nasal treatment of OXT increased c‐Fos expression in the PVN (472.8 ± 66.4/4 section), compared with control saline treatment (272.8 ± 52.4/4 section) (Figure 2C). The percentage of c‐Fos in OXTR‐positive neurons in OXT‐treated aged mice was significantly increased (53.4% ± 2.6%) compared with saline treated aged mice (29.8% ± 3.9%) (Figure 2D). The percentage of OXTR positive neurons in c‐Fos‐positive neurons was also increased in OXT‐treated aged mice (78.1% ± 3.5%) compared with saline‐treated aged mice (62.7% ± 2.5%) (Figure 2E). As to be expected, there was no change in the number of OXTR positive neurons between control (553.0 ± 20.7/4 section) and OXT‐treated (679.2 ± 68.0/4 section) groups (Figure 2F) 2 h postacute OXT administration. Imaging mass spectrometry visualization of a sagittal brain section of an aged WT mouse postacute nasal OXT treatment revealed extensive distribution of OXT throughout the brain when compared to a saline control (Figure S1). These data confirm that nasal OXT administration is highly effective and the administered OXT can be distributed within the brain and activate OXTR‐expressing neurons in the PVN.

Next, we examined the chronic effects of nasal treatment of OXT on aged mice. Nasal treatment of saline and OXT for 10 days had no effect on BW (Figure 2G), but increased plasma OXT levels (32.8 ± 7.7 pg/mL, saline: 14.4 ± 3.2 pg/mL) (Figure 2H). Furthermore, a decrease in the inflammation marker hs‐CRP was recorded (1.37 ± 0.04 μg/mL, control: 1.57 ± 0.06 μg/mL) (Figure 2I) to levels similar to that seen in young mice (1.33 ± 0.05 μg/mL) (Figure 1C).

When assessing the effect of nasal OXT treatment of aged mice on the mRNA levels of proteins playing key roles in secretion and epigenetic changes, chronic OXT treatment appears to rescue the effects of aging by decreasing Syt4 mRNA expression (Figure 2J), and increasing TET2 mRNA expression (Figure 2M) while having no effect on the expression of Sirt1, TET1, and TET3 mRNA (Figure 2K,L,N).

We next assessed the effect of a 10‐day OXT nasal treatment on the neurons of the hypothalamus and found that both OXT immunoreactive intensity and the number of OXT‐positive neurons were significantly increased (40.2 ± 4.2/section), compared with control saline‐injected mice (27.0 ± 3.3/section) (Figure 2O,P). Taken together, the data suggest that age‐related reduction of plasma OXT levels can be improved by nasal application of external OXT and may ameliorate aging‐related changes in molecular and cellular levels of OXT neurons.

### 2.3 The Expression of TET2 and COX IV in the Hypothalamus and Hippocampus of Young vs. Aged Mice

**FIGURE 3:**
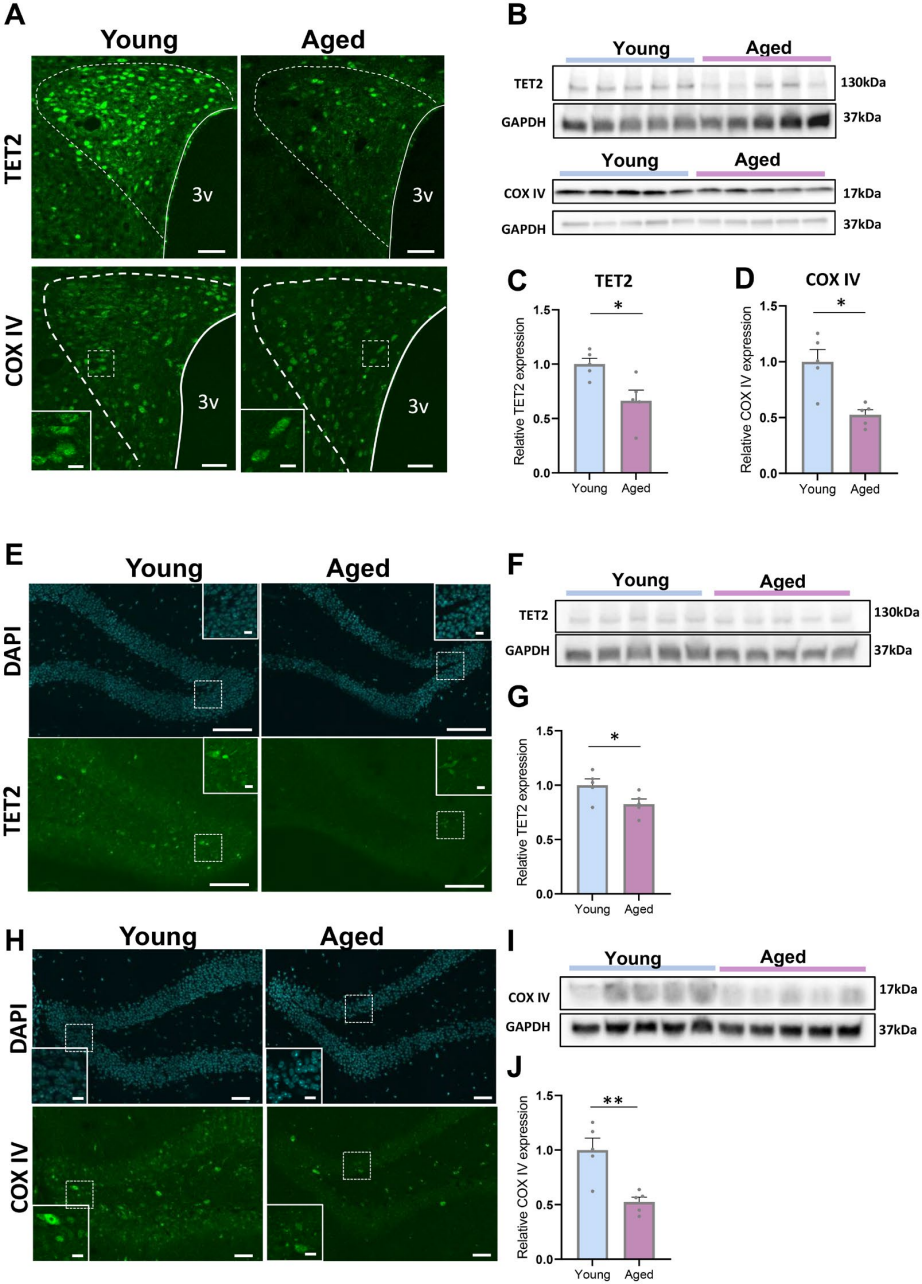
Expression of TET2 and COX IV in the hypothalamus and hippocampus of young versus aged mice. (A) Representative images of immunostaining of TET2 (upper panel) and COX IV (bottom panel) in young (9 weeks) and aged (62 weeks) mice of PVN. Scales = 50 μm. The panels located in the left bottom in COX IV staining are enlarged images of the dotted square in each image. Scales = 10 μm. (B) The image of western blotting of TET2 (upper panel) and COX IV (bottom panel) in young (9 weeks) and aged (62 weeks) mice of hypothalamus. (C, D) The relative expression of TET2 (C) and COX IV (D) to GAPDH in young and aged mice. The relative expressions of TET2 and COX IV in young mice are corrected to 1. *p < 0.05, unpaired *t*‐test, *n* = 5, 5. (E) Representative images of DAPI and immunostaining of TET2 in young (9 weeks) and aged (62 weeks) mice of the hippocampus. Scales = 50 μm. The right upper panels in each image are enlarged images of the dotted area in each image. Scale = 10 μm. (F) The image of western blotting of TET2 in young (9 weeks) and aged (62 weeks) mice of hippocampus. (G) The relative expression of TET2 to GAPDH in young and aged mice. The relative expressions of TET2 and COX IV in young mice are corrected to 1. *p < 0.05, unpaired *t*‐test, *n* = 5, 5. (H) Representative images of DAPI and immunostaining of COX IV in young (9 weeks) and aged (62 weeks) mice of the hippocampus. Scales = 50 μm. The bottom left panels in each image are enlarged images of the dotted area in each image. Scale = 10 μm. (I) The image of western blotting of COX IV in young (9 week) and aged (62 weeks) mice of hippocampus. (J) The relative expression of COX IV to GAPDH in young and aged mice. The relative expressions of COX IV in young mice are corrected to 1. *p < 0.05, unpaired *t*‐test, *n* = 5, 5.

Because TET2 deficiency is linked to increased mitochondrial dysfunction (Liu et al. 2020), we next examined the protein expression patterns of TET2 together with the mitochondrial respiratory complex IV (COX IV) in young and aged mice. In agreement with the mRNA expression results (Figure 1H), TET2 protein expression was significantly decreased in the hypothalamus, including PVN, in aged mice (Figure 3A upper panel, B,C) when compared to young mice. Expression of COX IV protein levels was also significantly decreased in the hypothalamus of aged mice (Figure 3A bottom panel, B,D). Similar to the hypothalamus, TET2 (Figure 3E–G) and COX IV protein (Figure 3H–J) were both significantly decreased in the hippocampus of aged mice.

To further confirm the functional implications of TET2 activity, staining of 5hmC, which is a TET2 activity reporter, was performed in young and aged mice (Figure S2). As shown in Figure S2, both the number of 5hmC positive neurons in the PVN and the intensity of 5hmC fluorescence in the dentate gyrus of the hippocampus were significantly reduced in aged mice.

The data indicate that aging decreases the expression of mitochondrial COX IV and TET2 protein and decreases DNA demethylation.

### 2.4 Sub‐Chronic Nasal Administration of OXT Rescues TET2 and COX IV Protein Expression in the Hypothalamus and Hippocampus of Aged Mice

**FIGURE 4:**
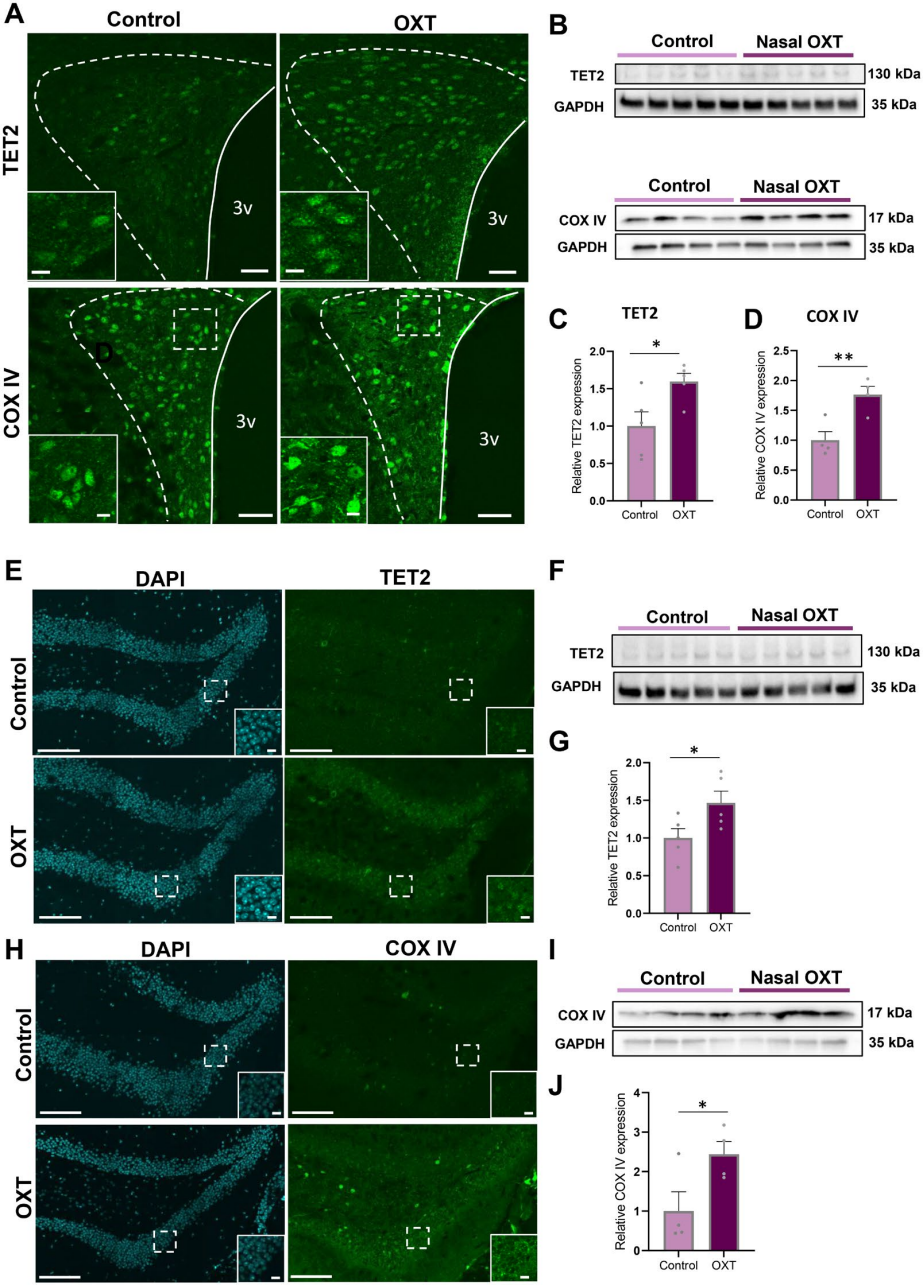
Impact of subchronic nasal OXT treatment on the expression of TET2 and COX IV in the hypothalamus and hippocampus of aged mice. (A) Representative images of immunostaining of TET2 (upper panel) and COX IV (bottom panel) in control and OXT treated aged mice (62 weeks) of PVN. Scales = 50 μm. The panels located in the left bottom in COX IV staining are enlarged images of the dotted square in each image. Scales = 10 μm. (B) The image of western blotting of TET2 (upper panel) and COX IV (bottom panel) in control and OXT treated aged mice (62 weeks) of hypothalamus. (C, D) The relative expression of TET2 (C) and COX IV (D) to GAPDH in control and OXT treated aged mice. The relative expressions of TET2 and COX IV in control mice are corrected to 1. *p < 0.05, unpaired *t*‐test, *n* = 4–5. (E) Representative images of DAPI and immunostaining of TET2 in control and OXT treated aged mice (62 weeks) of hippocampus. Scales = 50 μm. The bottom right panels in each image are enlarged images of the dotted area in each image. Scale = 10 μm. (F) The image of western blotting of TET2 in control and OXT treated aged mice (62 weeks) of hippocampus. (G) The relative expression of TET2 to GAPDH in young and aged mice. The relative expressions of TET2 in control mice are corrected to 1. *p < 0.05, unpaired *t*‐test, *n* = 5, 5. (H) Representative images of DAPI and immunostaining of COX IV in control and OXT treated aged mice (62 weeks) of hippocampus. Scales = 50 μm. The bottom right panels in each image are enlarged images of the dotted area in each image. Scale = 10 μm. (I) The image of western blotting of COX IV in control and OXT treated aged mice (62 weeks) of hippocampus. (J) The relative expression of COX IV to GAPDH in control and OXT treated aged mice. The relative expressions of COX IV in control mice are corrected to 1. *p < 0.05, unpaired *t*‐test, *n* = 4–5.

Consistent with mRNA expression results (Figure 2M), TET2 protein expression was significantly increased (Figure 4A–C). COX IV expression also significantly increased in the hypothalamus of aged mice in response to subchronic administration (10 days) of OXT treatment (Figure 4A bottom, B bottom, D). Similarly, the increased protein expression of both TET2 and COX IV was also confirmed in the aged murine hippocampus, following subchronic nasal OXT treatment (TET2: Figure 4E–G) (COX IV: Figure 4H–J).

In order to examine DNA demethylation status, 5hmC staining was performed in aged mice after 10 days of nasal OXT treatment. The number of 5hmC‐positive neurons and the intensity of the 5hmC fluorescence signal in the dentate gyrus of the hippocampus were dramatically increased in OXT‐treated aged mice (Figure S3), confirming a functional impact of the increased TET2 levels reported. The data would suggest the possibility that nasal OXT administration enhances the protein expression of mitochondrial COX IV in aged mice of the hypothalamus and hippocampus via the recovery of TET2 activity and subsequent DNA demethylation over time.

### 2.5 OXTR Mediated Pathway for Regulation of the TET2 and COX IV Expression

Next, in order to examine the impact of endogenous OXT on TET2 and COX IV, protein expression levels were compared in the hypothalamus and hippocampus of aged WT vs. aged OXTR null mice.

**FIGURE 5:**
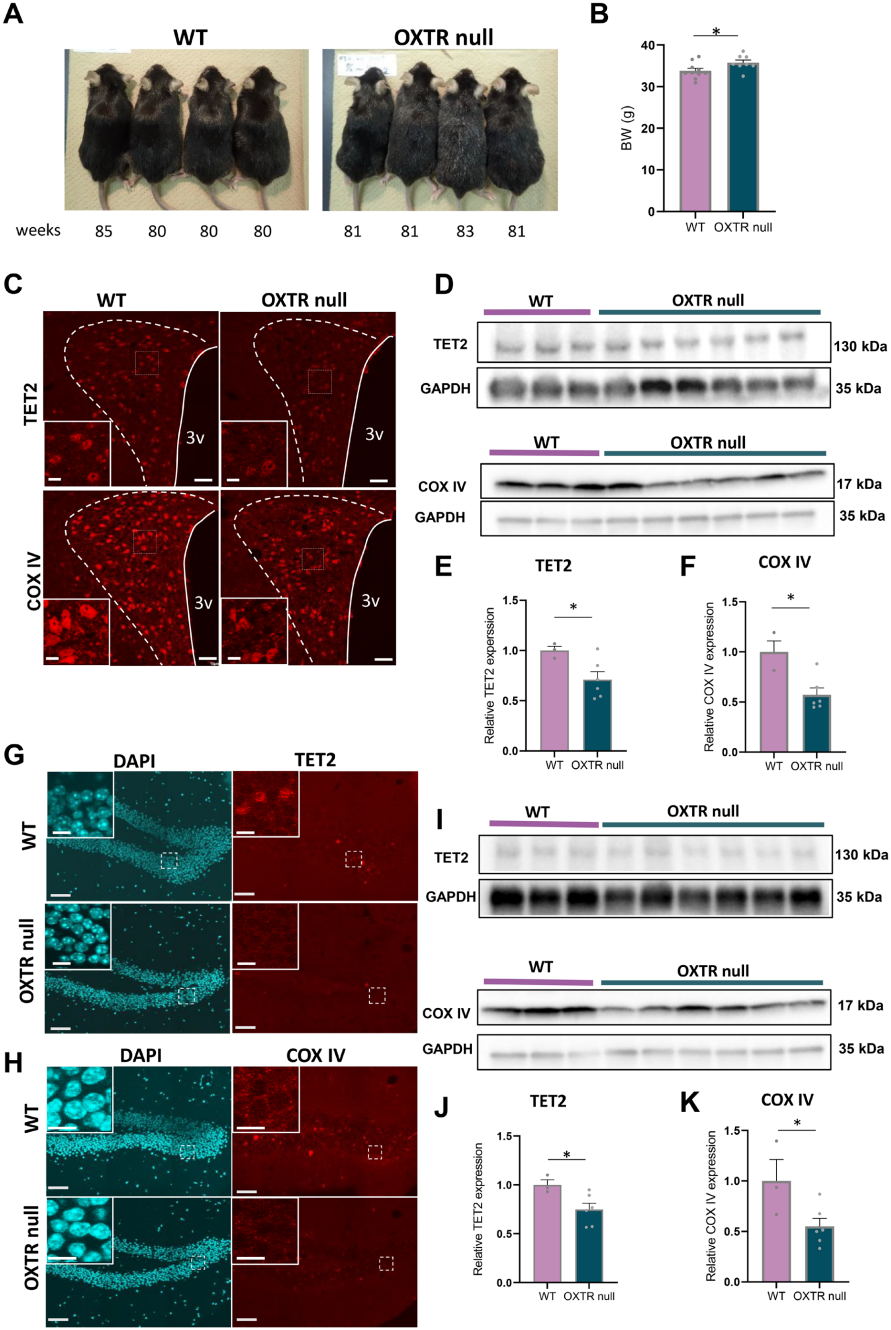
Expression of TET2 and COX IV in aged WT and aged OXTR null mice. (A) The appearance of aged WT (80–85 weeks old) and OXTR null mice (81–83 weeks old). The number below each mouse indicates the weeks age. (B) Comparison of BW between aged control (80–85 weeks) and OXTR null (81–89 weeks) mice. *p < 0.05, unpaired *t*‐test, *n* = 10, 8. (C) Representative images of immunostaining of TET2 (upper panel) and COX IV (bottom panel) in WT (84 weeks) and OXTR null (84 weeks) mice of PVN. Scales = 50 μm. The left bottom panels in each image are enlarged images of the dotted area in each image. Scale = 10 μm. (D) The image of western blotting of TET2 (upper panel) and COX IV (bottom panel) in aged WT (80–85 weeks) and OXTR null mice (81–89 weeks) of hypothalamus. (E, F) The relative expression of TET2 (E) and COX IV (F) to GAPDH in aged WT and OXTR null mice. The relative expressions of TET2 and COX IV in aged WT mice are corrected to 1. *p < 0.05, unpaired *t*‐test, *n* = 3, 6. (G, H) Representative images of DAPI and immunostaining of TET2 (G) and COX IV (H) in aged WT (84 weeks) and OXTR null (84 weeks) mice of hippocampus. Scales = 50 μm. The panels located at the left corner in each image are enlarged images of the dotted area in each image. Scale = 10 μm. (I) The image of western blotting of TET2 (upper panel) COX IV (bottom panel) in aged WT (80–85 weeks) and OXTR null mice (81–89 weeks) of hippocampus. (J, K) The relative expression of TET2 (J) and COX IV (K) to GAPDH in aged WT and OXTR null mice. The relative expressions of TET2 and COX IV in aged WT mice are corrected to 1. *p < 0.05, **p < 0.01, unpaired *t*‐test, *n* = 3, 6.

As shown in Figure 5A, the appearance of aged WT and aged OXTR null mice (80–85 weeks old) was visibly different. Even though the images were shown under the same optical conditions, gray hair and loss of a glossy, healthy coat appearance were clearly visible in OXTR null aged mice, compared with similarly aged WT mice. OXTR null mice also weighed 5.9% more than WT mice of a similar age (Figure 5B).

Protein expression of TET2 and COX IV in the hypothalamus and hippocampus was significantly decreased in aged OXTR null mice compared with aged WT mice (PVN: Figure 5C–F, hippocampus: Figure 5G–K).

DNA demethylation status was examined by fluorescent visualization of 5hmC in aged OXTR null mice. Both the number of 5hmC‐positive neurons and the intensity of 5hmC fluorescence in the dentate gyrus of the hippocampus were significantly lower in OXTR null mice, compared with aged WT mice (Figure S4).

This data further supports our data to suggest that endogenous OXT and OXTR activity promote COX IV expression via increased TET2 expression and DNA demethylation.

### 2.6 The Impact of OXT Administration on the Aged Primary Neurons

**FIGURE 6:**
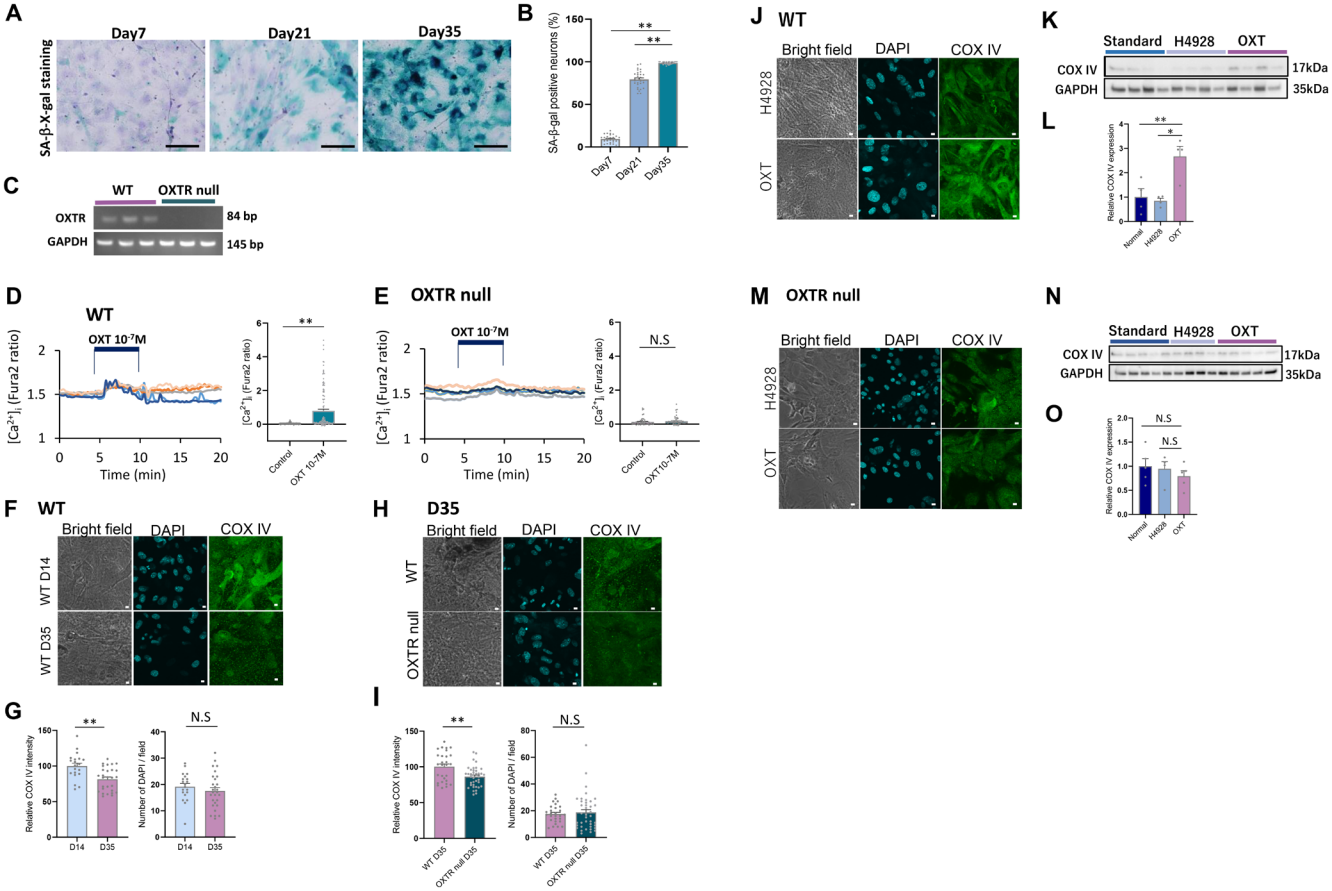
Impact of OXT on aged primary neurons. (A) Senescence‐associated beta‐galactosidase (SA‐β‐X‐gal) staining in primary neurons cultured for 7, 21, and 35 days. (B) The percentage of SA‐β‐X‐gal positive neurons to total neurons. **p < 0.01, one‐way ANOVA followed by Tukey's multiple range test, *n* = 30 (field). (C) OXTR mRNA from primary neurons of WT and OXTR null mice. Although bands in the WT are expressed at 84 bp, the bands from OXTR null mice were completely absent. GAPDH (145 bp) was used to evaluate loading. (D) The change of cytosolic [Ca^2+^]_i_ in primary neurons from WT mice (left panel). The amplitude of [Ca^2+^]_i_ under treatment of 10^−7^ M OXT, compared with the amplitude of [Ca^2+^]_i_ pretreatment of OXT. **p < 0.01, paired *t*‐test, *n* = 130. (E) The change of [Ca^2+^]_i_ in primary neurons from OXTR null mice (left panel). The amplitude of [Ca^2+^]_i_ under treatment of 10^−7^ M OXT, compared with the amplitude of [Ca^2+^]_i_ pretreatment of OXT. ns; no significant differences, paired *t*‐test, *n* = 55. (F) Representative images of COX IV staining in primary neurons from WT at Day 14 (upper panels) and Day 35 (bottom panels). (G) The relative intensity of COX IV fluorescence intensity per field (left panel). **p < 0.01, unpaired *t*‐test, *n* = 30, 22 (field). The right panel indicates the number of DAPI in the intensity of COX IV analyzed in each field. ns; no significant differences, unpaired *t*‐test, *n* = 30, 22 (field). (H) Representative images of COX IV staining in primary neurons from WT (upper panels) and OXTR null (bottom panels) at Day 35. (I) The relative intensity of COX IV fluorescence intensity per field (left panel). **p < 0.01, unpaired *t*‐test, *n* = 19, 30 (field). The right panel indicates the number of DAPI in the intensity of COX IV analyzed in each field. ns; no significant differences, unpaired *t*‐test, *n* = 19, 30 (field). (J) The representative images of COX IV staining in primary neurons from WT, treated with OXTR antagonist (10^−7^ M H4928, upper panels) and OXT (10^−9^ M, bottom panels) for 7 days. Each treatment was started at Day 35 and sampling was performed at Day 43. (K) The image of western blotting of COX IV in primary neurons from WT, treated with H4928, OXT, or nontreated standard medium. (L) The relative expression of COX IV to GAPDH in primary neurons from WT, treated with H4928, OXT, or nontreated. COX IV in nontreated neurons is corrected to 1. *p < 0.05, **p < 0.01, one‐way ANOVA followed by Tukey's multiple range test, *n* = 4 (plate). (M) The representative images of COX IV staining in primary neurons from OXTR null mice, treated with 10^−7^ M H4928 (upper panels) and OXT (10^−9^ M, bottom panels) for 7 days. Each treatment was started at Day 35 and sampling was performed at Day 43. (N) The image of western blotting of COX IV in primary neurons from OXTR null mice, treated with H4928, OXT, or nontreated standard medium. (O) The relative expression of COX IV to GAPDH in primary neurons from OXTR null mice, treated with H4928, OXT, or nontreated. COX IV in nontreated neurons is corrected to 1. ns; no significant differences, one‐way ANOVA followed by Tukey's multiple range test, *n* = 5 (plate).

To examine the direct effect of OXT on neurons through OXTR signaling pathways, primary neurons were established from fetal brain. To confirm the aging stages in the primary neuron cultures, detection of the senescence marker, senescence‐associated β‐galactosidase (SA‐β‐gal) (Childs et al. 2019), was performed using SA X‐gal staining at 7, 21, and 35 days after establishing the culture. The SA X‐gal staining revealed that almost all primary neurons (98.6% ± 0.3%) expressed SA‐β‐gal after 35 days of primary cell culture (Figure 6A,B). Thus, in this study, we defined primary neurons cultured over 35 days as a model of “aged” primary neurons, while primary neurons cultured only for 14 days were defined as a model of “young” primary neurons.

In cultured primary neurons, the expression of OXTR was confirmed in WT and absent in OXTR null mice primary neurons (Figure 6C). This was further confirmed functionally by investigating the activation of OXTR in primary neurons by measuring cytosolic Ca^2+^ ([Ca^2+^]_i_). A 10^−7^ M OXT treatment significantly increased [Ca^2+^]_i_ in primary neurons from WT (Figure 6D); whereas no changes in [Ca^2+^]_i_ were observed in primary neurons from OXTR null mice (Figure 6E). These results indicate that functionally active OXTR is expressed in primary neurons from WT mice.

By analyzing optical brightness per visual field for COX IV protein expression, protein expression was significantly decreased in aged primary neurons, compared with young neurons (Figure 6F). While the DAPI‐positive cell number was not affected (Figure 6G right), aged neurons showed a significant decrease in COX IV fluorescence intensity (Figure 6G left). The COX IV‐positive intensity of primary neurons was significantly decreased in aged WT and aged OXTR null mice, while the DAPI‐positive cell number was not altered (Figure 6H,I).

Next, to examine the chronic effect of OXT on COX IV expression in aged primary neurons from WT mice, aged neurons were cultured in standard medium, 10^−9^ M OXT containing medium, or 10^−7^ M H4928 (OXTR antagonist) containing medium for 7 days.

It is important to note that culture medium contains 5% fetal bovine serum as well as horse serum, and it is therefore impossible to avoid the influence of endogenous OXT in fetal bovine serum and horse serum. When measured, the culture media contained 20.7 ± 2.8 pg/mL (approximately 2 × 10^−11^ M) OXT. Therefore, the OXTR antagonist, H4928, was added to evaluate the effect of added OXT alone.

OXT treatment for 7 days enhanced the expression of COX IV, compared with H4928 treated neurons (Figure 6J), and COX IV protein in OXT‐treated neurons was significantly increased compared with that in neurons cultured in standard medium or H4928 containing medium (Figure 6K,L). However, there was no difference in the expression of COX IV between primary neurons cultured in standard medium and H4928 containing medium (Figure 6K,L). To confirm the DNA methylation status, 5hmC immunocytochemistry was performed on aged WT neuronal cultures treated with H4928 or OXT for 7 days (Figure S5). OXT treatment significantly increased 5hmC fluorescence intensity when compared to H4928‐treated cultures, further confirming a role for OXTR signaling; there were no changes in COX IV expression in primary neurons from OXTR null mice between standard, H4928, or OXT treated groups (Figure 6M–O) in clear contrast to WT OXT‐treated cultures (Figure 6J–L).

These results strongly suggest that OXT enhances COX IV expression in primary neurons via direct activation of OXTR and subsequent demethylation of DNA.

### 2.7 The Impact of OXT/OXTR on O‐GlcNAc Transferase (OGT)

OGT has been identified as a key cofactor of TET2 activity (Chen et al. 2013). To investigate the direct effects of OXT on OGT, OGT expression was compared in primary neurons from WT mice treated with standard medium, H4928, and OXT containing medium (Figure S6A,B). OXT treatment of aged primary neuronal cultures significantly increased OGT expression compared with the standard medium. However, no differences in OGT expression were observed in primary neurons from OXTR null mice (Figure S6C,D). In addition, decreased expression of OGT was found in the hippocampus of aged mice compared with young mice (Figure S6E,F). Nasal administration of OXT increased OGT expression in the aged hippocampus (Figure S6G,H). Furthermore, OGT expression was significantly decreased in the hippocampus of aged OXTR null mice compared with aged WT mice (Figure S6I,J). These findings suggest that OXT enhances the expression of OGT, as well as TET2, in aged mice via OXTR signaling cascades.

## 3 Discussion

The antiaging effects and system‐wide health benefits of the neuropeptide OXT in humans have been known for decades (Carter et al. 2020). Past reports also highlight a decline of plasma OXT levels with aging in mammals (Elabd et al. 2008, 2014). In this study, we present a novel mechanism underpinning the age‐related decrease in circulating OXT levels, as well as the identification of a previously unknown epigenetic effect of nasal OXT administration that promotes mitochondrial health.

Our current study demonstrates that the decline of plasma OXT levels with aging is attributable to two primary mechanisms. Firstly, aging results in a reduction in the number of OXT‐positive neurons. Secondly, aging leads to an increased production of Syt4, which acts as a negative regulator of OXT secretion. These two factors together decrease plasma OXT levels in mice over 45 weeks old in this study.

In line with previous reports, our results would indicate that the accumulation of methylation with age plays a significant role in the observed decrease in OXT‐positive neurons in aged mice. Usmani et al. reported that age‐related DNA methylation induces a decline in OXT mRNA levels by 50% in the hypothalamus of aged mice (15‐month‐old), compared to young mice (2‐month‐old) (Usmani et al. 2024). The TET family, TET1, TET2, and TET3, is known to play a critical role in DNA demethylation through a series of oxidative reactions (Zhang et al. 2023). TET enzymes regulate demethylation by catalyzing the conversion of 5mC to 5hmC, then to 5fC, and finally to 5caC (Zhang et al. 2023). Our study shows that TET family expression was significantly decreased or tended to be decreased in the hypothalamus of aged mice. Our data support the hypothesis that aging diminishes the production of OXT by reducing the expression of TET, thereby promoting DNA methylation and silencing gene expression, which ultimately leads to a reduction in OXT production. The observed decrease in 5hmC positive neurons in the hypothalamus and hippocampus of aged mice (Figure S2), and previous reports (Usmani et al. 2024) are in agreement with this hypothesis.

TET2 is extensively expressed in neurons, particularly in the cerebral cortex and hippocampus, and it plays a vital role in sustaining neuronal survival (Mi et al. 2015). The TET family influences mitochondrial biogenesis by regulating genes involved in oxidative phosphorylation and mitochondrial quality control (Leon Kropf et al. 2024). Knockdown of TET2 accompanied by reduction of 5hmC induced mitochondrial dysfunction and apoptosis in hippocampal neurons (Liu et al. 2020). Consistent with these reports, our results showed that aged mice exhibited the decrease of both TET2 and COX IV expression (Figure 3); that may lead to the decline in mitochondrial function.

Of interest to note, the recorded decrease in TET2 expression (hypothalamus; 33.6% ± 9.9%, hippocampus; 17.4% ± 4.6% decline in aged mice) is less than that of COX IV expression (hypothalamus; 45.7% ± 8.1%, hippocampus; 52.4% ± 4.5% decline in aged mice) (Figure 3). Previous studies have highlighted the role of O‐GlcNAc transferase (OGT) in demethylation, serving as a key cofactor of TET2 activation (Chen et al. 2013; Deplus et al. 2013). Consistent with the report by Wheatley et al. (2019), our findings also demonstrated decreased OGT expression in the hippocampus of aged mice (Figure S6E,F). Moreover, restoring neural OGT in the aged hippocampus has been reported to rejuvenate cognition (Wheatley et al. 2019); we show that nasal administration of OXT increased OGT expression in the hippocampus of aged mice (Figure S6G,H). Taken together, our data suggest that the synergistic effects of OXT‐mediated TET2 and OGT expression may promote demethylation and protect against the detrimental effects of aging. Much work remains to be done to further elucidate the underlying synergistic mechanisms involving OXT, TET2, and OGT action in neurons.

Syt4 is a member of the synaptotagmin family of proteins. Synaptotagmins constitute a large family and are thought to be involved in both regulated and constitutive vesicular trafficking (Mikoshiba et al. 1999). Syt4 is known to be expressed in both the brain and neuroendocrine system (Vician et al. 1995). Zhang et al. reported that Syt4 is predominantly expressed in OXT neurons in the PVN and negatively regulates OXT exocytosis (Zhang and Cai 2011). Similarly, CD38, a membrane glycoprotein with ADP‐ribosyl cyclase activity, has been known as a key factor in the OXT secretion process (Jin et al. 2007). However, as no changes in CD38 mRNA expression were observed in aged mice (Figure 1E), the age‐related increase in Syt4 expression is most likely the mechanism responsible for the reduction in plasma OXT levels.

Previous work from our group demonstrated that the firing frequency of action potentials in the PVN OXT neurons was age‐dependently increased as a result of a loss of inhibitory input from NPY neurons in the arcuate nucleus (Maejima et al. 2019). It is known that Syt4 mRNA and protein levels are regulated by neuronal activity (Barber et al. 2009) and Syt4 expression may therefore be regulated by multiple factors including age‐dependent neuronal activation, as well as DNA methylation. The exact underlying mechanism which drives Syt4 expression, therefore, remains to be elucidated.

OXT is known for its poor blood–brain barrier permeability (0.002%) and brief half‐life (19 min) (Mens et al. 1983). Nasal administration of OXT has been shown to be an effective noninvasive route of administration. A series of studies investigated the pharmacokinetics of OXT following nasal application, consistently reporting significant increases in OXT levels in both plasma (with peaking 15–30 min, persisting for 75–90 min) and cerebrospinal fluid (CSF) (with peaking 15–75 min) (Freeman et al. 2016; Lee et al. 2018; Neumann et al. 2013). Since nasal treatment of OXT increases peripheral OXT levels, it is possible that peripheral OXT activates OXTR in the vagus nerve (Iwasaki et al. 2015) subsequently activating the ascending neural pathway from the nucleus solitary tract (NTS) to the PVN (Maejima et al. 2011, 2015), ultimately inducing the release of OXT from PVN OXT neurons and further enhancing OXT release in an autocrine/paracrine manner (Carson et al. 2010; Zhang and Cai 2011). Although exogenous OXT treatment stimulates endogenous OXT secretion, it is challenging to distinguish between exogenously applied and endogenously secreted OXT. However, using OXT deficient mice, Smith et al. demonstrated for the first time that after nasal or intraperitoneal administration of OXT, its concentration in both peripheral circulation and extracellular brain fluid (amygdala and dorsal hippocampus) rose within the first 30 min of administration and remained elevated for the subsequent hour (Smith et al. 2019). Our data also confirmed that nasal OXT treatment increased plasma OXT levels in OXT null mice at 15 min posttreatment (Figure 2B). Our data, along with the previous report, indicates that exogenously administered OXT can reach both the peripheral circulation and the brain. As shown in Figure 2A, OXTRs are abundantly distributed in the PVN. Our previous study showed that approximately 53% of OXT neurons express OXTR (Maejima et al. 2021). In addition, MS imaging demonstrated that a single nasal OXT treatment distributed OXT into the brain, especially in the brainstem regions (Figure S1). These results presented here are therefore in agreement with previous pharmacokinetics studies and suggest that the activation of OXTR signaling is indeed driving the observed changes reported here following nasal treatment of OXT for 10 days.

To further confirm the importance of OXT‐OXTR signaling in neurons, we examined TET2 and COX IV expression in aged OXTR null mice and found that both TET2 and COX IV expression in the hypothalamus and hippocampus were indeed decreased in OXTR null mice (Figure 5). This study provides the first comprehensive investigation of the relationship between OXT and TET2, advancing our understanding of the mechanism for OXT's antiaging effect. Consistent with our present results, genome‐wide 5hmC profiles in the hippocampus of TET2 neuron‐specific KO (TET2 CKO) mice, combined with high‐throughput sequencing, revealed that the TET2 CKO‐induced differentially hydroxymethylated regions (DhMRs) were significantly enriched in the OXT signaling pathway as well as in the glutamatergic synapse and circadian entrainment (Q. Zhang et al. 2021).

Another critical factor in the aging process is the dysfunction of mitochondria. OXT exhibits antioxidant and anti‐inflammatory properties that can counteract neuroinflammation and mitochondrial dysfunction associated with aging and depression (Amini‐Khoei et al. 2017; Bordt et al. 2019). Therefore, OXT holds promise as a treatment for enhancing mitochondrial function and thereby mitigating aging.

Methylation plays a crucial role in the aging process as well as in pathology, acting as an epigenetic mechanism that influences gene expression without altering the DNA sequence. This relationship is highlighted by the concept of “epigenetic clocks”, which use DNA methylation patterns to predict biological age and assess the risk of age‐related diseases (Noroozi et al. 2021). DNA methylation changes accumulated during aging disrupt homeostatic processes, contributing to chronic inflammatory states and the progression of age‐related diseases such as cancer, autoimmunity, metabolic disorders, hematologic neoplasms, and solid tumors (Younesian et al. 2024). Therefore, the maintenance and regulation of the demethylation mechanism is important for preventing the progression of age‐related diseases. The present study demonstrated that OXT promotes demethylation via increased TET2 expression in neurons. This strongly suggests that OXT may function as an antiaging agent and also has the potential to ameliorate age‐related neurological disorders.

Although this study focused on the effects of OXT only on neurons, nasal administration of OXT restored plasma OXT levels and decreased CRP levels (Figure 2I), a marker of systemic inflammation. Factors secreted by senescent cells, known as the senescence‐associated secretory phenotype (SASP), such as IL‐6 and TNFα, promote chronic inflammation (Li et al. 2023). SASP increases CRP levels, and it is reported that CRP levels reflect the effects of SASP, especially IL‐6 (Puzianowska‐Kuźnicka et al. 2016). Therefore, systematic inflammation and an increase in CRP levels are acknowledged as key indicators of aging and age‐associated diseases (Tang et al. 2017). Recent studies revealed that peripheral administration of OXT decreased CRP levels in inflammatory models (Ahmed and Elosaily 2011). Since OXTR is expressed in various tissues, including hepatocyte and immune cells (Luo et al. 2021; Szeto et al. 2017), it is considered that OXT exerts an anti‐inflammatory effect on a wide range of peripheral tissues. Because plasma CRP levels were decreased with nasal administration of OXT, it indicates that restoring serum OXT levels by nasal treatment of OXT is effective enough to reduce systemic inflammation.

Taken together, our data suggest an OXT‐TET‐DNA demethylation cycle to explain the protective effect of nasally administered OXT (Figure S7). DNA methylation, mitochondrial dysfunction, inflammation, and senescence interact to form a vicious cycle (Miwa et al. 2022; Walker et al. 2022). The decline in TET2 expression due to aging increases methylation and mitochondrial dysfunction. Methylation levels in hypothalamic neurons decrease OXT production and decrease plasma OXT levels. The decline in plasma OXT levels and mitochondrial dysfunction together promote systemic inflammation, which accelerates senescence. However, nasal administration of OXT may interrupt this vicious cycle by increasing TET2 expression, inducing DNA demethylation, and raising plasma OXT levels.

Plasma OXT levels increase three‐ to four‐fold at birth in humans (Uvnäs‐Moberg et al. 2019). Following parturition, pulsatile OXT secretion is induced in response to breastfeeding, facilitating milk ejection (Uvnäs Moberg et al. 2020). Research on DNA methylation‐based epigenetic clocks indicated that pregnancy accelerates aging by approximately 2 years. However, some participants exhibited a younger biological age at postchildbirth (Pham et al. 2024). Additionally, a systematic review and meta‐analysis revealed that women who breastfeed have a lower risk of ovarian cancer and breast cancer (Tschiderer et al. 2022). These findings suggest that OXT secretion during parturition and breastfeeding may delay biological aging, further supporting our findings of the close links between OXT, DNA methylation status, and aging.

This study, however, has several limitations. Due to the logistical limitations of keeping aged animals, the experiments reported here were conducted using a relatively small number of animals; data should therefore be interpreted with caution. Furthermore, only male animals were used in this study. The female steroid hormone, estrogen, has various antiaging functions and is vascular protective, neuroprotective, and has antioxidant activity (Baltgalvis et al. 2010; Knowlton and Lee 2012; Russell et al. 2019). In order to specifically investigate the effects of exogenous OXT on aging and mitochondrial function, we used only male animals in order to minimize the additional complications of estrogen action in this study. Further research on females and additional animal numbers is needed to strengthen the conclusions drawn by this study.

## 4 Conclusion

Here, we report that the nasal administration of OXT suppresses systemic inflammation in aged mice and promotes DNA demethylation via TET2 activity and the subsequent enhanced expression of mitochondrial COX IV in neurons. Our results presented here suggest that OXT may exert its antiaging effects through mechanisms that involve multiple systems and may be a powerful tool to help prevent aging in neurons.

## 5 Materials and Methods

### 5.1 Animals

Nine‐ to 12‐week‐old male C57BL/6J mice (purchased from Japan SLC (Japan)), OXTR deficient (OXTR null) mice (Takayanagi et al. 2005) and OXT deficient (OXT null) mice (Nishimori et al. 1996) were used in this study. Animals were maintained on a 12 h light/dark cycle (Turn on, 7:00; Turn off, 19:00), were housed in individual cages, and fed a standard diet (CE‐2: Clea, Osaka, Japan). Aged mice (WT and OXTR null) were kept in animal facilities until they reached the desired age. Since one human year is almost equivalent to nine mice days when correlating their entire lifespan (Dutta and Sengupta 2016) “aged” was defined as over 45 weeks old, which corresponds to 35 years old in humans in this study.

OXT receptor (oxtr)^VenusΔNeo/+^, which exhibits venus fluorescence in OXTR‐expressing cells (OXTR‐Venus) (Yoshida et al. 2009); male mice were used for histochemical analysis of OXTR. Experimental procedures and care of animals were conducted according to the Fukushima Medical University Institute of Animal Care and Use Committee. This study protocol was reviewed and approved by Fukushima Medical University Institute of Animal Care and Use Committee, approval number 2023022, 2023021.

### 5.2 Quantitative Real‐Time PCR

Nine weeks and 46–47‐weeks‐old male mice were anesthetized (10 mL/kg), intraperitoneal injection of a mixture of three anesthetic agents (composition; Medetomidine [0.003%, Domitor, Nippon Zenyaku Kogyo Co. Ltd., Koriyama, Japan], midazolam [0.04%, Dormicum, Astellas Pharma Inc., Tokyo, Japan], and butorphanol tartrate [0.05%, Vetorphale, Meiji Seika Pharma Co. Ltd., Tokyo, Japan]); and blood and brain were collected. Brain tissues were sectioned (0.14 mm to −2.06 mm from bregma), and the hypothalamus was further dissected from sections. Total RNA was isolated from the hypothalamus using the RNeasy Mini Kit (QIAGEN, Hilden, Germany) and Monarch RNA Purification Columns (New England BioLabs Inc., MA). cDNA synthesis was performed using an M‐MLV (Thermo Fisher Scientific, MA), RNaseOUT Recombinant Ribonuclease Inhibitor (Thermo Fisher Scientific, MA), and dNTP (Agilent Technologies, TX). A quantitative RT‐PCR assay was performed using the TB Green Premix Ex Taq II (Tli RNaseH Plus, Takara Bio Inc., Shiga, Japan). The cycling protocol was: initial denaturation at 95°C for 30 s, then 35 cycles of 95°C for 5 s, 56°C for 10 s, then 72°C for 15 s. Product accumulation was measured in real time, and the mean cycle thresholds were determined. Expression levels of each mRNA were calculated using the 2^ΔΔCT^ method of relative quantification and normalized to the housekeeping gene GAPDH. The PCR primers are shown in Table 1.

**TABLE 1:** Primers used in this study.

Target mRNA	Forward primer	Revere primer	bp	Accession number
Syt4	ATGGCTCCTATCACCACCAG	AGCAGATCCAGGCAAAGAGA	112	NM_031693
CD38	CGCTGCCTCATCTACACTCA	TTGCAAGGGTTCTTGGAAAC	107	NM_007646
Sirt‐1	CGGCTACCGAGGTCCATATAC	CAGCTCAGGTGGAGGAATTGT	109	NM_001159589
TET1	GAGCCTGTTCCTCGATGTGG	CAAACCCACCTGAGGCTGTT	202	NM_001253857
TET2	TGTTGTTGTCAGGGTGAGAATC	TCTTGCTTCTGGCAAACTTACA	103	NM_001040400
TET3	CCGGATTGAGAAGGTCATCTAC	AAGATAACAATCACGGCGTTCT	162	NM_001347313
OXTR	GTGCAGATGTGGAGCGTCT	GTTGAGGCTGGCCAAGAG	84	NM_001081147
GAPDH	TCCACTCACGGCAAATTCAACG	TAGACTCCACGACATACTCAGC	145	NM_001289726

### 5.3 The Measurement of Plasma Hs‐CRP

Blood samples were collected from 9 to 11 (young) and 47–51 (aged) weeks old mice into EDTA tubes and centrifuged at 3000 rpm for 10 min at 4°C. Plasma was collected, and plasma CRP concentrations were measured by mouse hs‐CRP ELISA kit (KT‐095, Kamiya Biomedical Company, WA). Intra‐assay and interassay variations were under 10%.

### 5.4 The Measurement of Plasma OXT

Blood samples were collected from 9 to 11 (young) and 47–51 (aged) male mice into EDTA and aprotinin‐containing tubes, and plasma was collected. Before the measurements, 0.1 M HCl was added to the supernatant, and OXT was recovered by a C18 Sep‐Pak column (Waters Co., MA) and was extracted with methanol. OXT concentration was measured using the OXT EIA kit (Enzo Life Sciences/Assay Designs, NY). Intra‐assay and interassay variation were 12.6%–13.3% and 11.8%–20.9%, respectively.

### 5.5 Immunostaining OXT, COX IV, TET2

Mice were intraperitoneally injected with a mixture of three types of anesthetic agents (10 mL/kg) and perfused intracardially with 4% paraformaldehyde (PFA) and 0.2% picric acid. Serial coronal sections (40 μm) were collected from each mouse using a freezing microtome. The sections were washed in PBS and incubated with 0.1% Triton‐X for 30 min. In order to block autofluorescence, True Black (#23007, Biotium, CA) was treated for 5 min. The sections were washed in PBS and incubated for 1 h in a blocking solution comprising 2% bovine serum albumin (BSA) and 5% normal goat serum (NGS). Sections were incubated with mouse antioxytocin mouse monoclonal antibody (1:1000; MAB5296, Merck Millipore, MA) or anti‐COX IV rabbit polyclonal antibody (1:500; PM063, MBL, Tokyo, Japan) or anti‐TET2 rabbit polyclonal antibody (1:500; 21,207‐1‐AP, proteintech, IL) in blocking solution overnight at 4°C. Then sections were incubated with Alexa fluor 594‐labeled goat antimouse IgG (1:400; Life Technologies, CA) or Alexa fluor 488‐labeled goat antirabbit IgG (1:400; Life Technologies, CA) or Alexa fluor 594‐labeled goat antirabbit IgG (1:400; Life Technologies, CA) for 40 min. Sections were mounted on glass slides and covered. Confocal fluorescence images of PVN or dentate gyrus of hippocampus were acquired by FV10i (Olympus, Tokyo, Japan) under similar light conditions. OXT‐positive cells in PVN were counted per section.

### 5.6 Nasal Treatment of OXT and Double Staining c‐Fos and OXTR

Forty‐seven to 50 weeks old OXTR‐Venus were habituated for 10 days in individual cages. Two hours before the experiment, food was removed from each cage. According to our previous study (Maejima et al. 2015), 10 μg/10 μL of OXT (4084‐v, Peptide Institute, Osaka, Japan) or vehicle (0.9% saline) was dropped into the nasal cavity. The dose (10 μg) of OXT was an enough dose to affect food intake regulation via the central nervous system (Maejima et al. 2015). Two hours after nasal treatment with OXT or saline, mice were intraperitoneally injected with a mixture of three types of anesthetic agents (10 mL/kg) and perfused intracardially with 4% PFA and 0.2% picric acid. Serial coronal sections (40 μm) were collected from each mouse using a freezing microtome. The sections every 160 μm were used for immunostaining. The sections were washed in PBS and incubated for 1 h in a blocking solution containing 0.1% Triton‐X‐100, 2% BSA, and 5% NGS. Sections were incubated with chicken anti‐GFP polyclonal antibody (1:500; ab13970, abcam, Cambridge, UK) and with rabbit anti‐c‐Fos polyclonal antibody (1:1000; RPCA‐c‐Fos AP, Encor Biotechnology Inc., FLA) in blocking solution overnight at 4°C. Then, sections were incubated with Alexa flour 488‐labeled goat antichicken antibody (1:500; Life Technologies, CA) and Alexa flour 594‐labeled goat antirabbit antibody (1:500; Life Technologies, CA) for 40 min. Sections were mounted on glass slides and covered. Confocal fluorescence images of PVN were acquired by FV10i (Olympus, Tokyo, Japan) under similar light conditions. GFP and c‐Fos‐positive cells in PVN were counted per section.

### 5.7 Nasal Treatment of OXT‐to‐OXT Null Mice

Male OXT null mice, aged 12–16 weeks (Nishimori et al. 1996) were nasal treated with either saline or OXT (10 μg/10 μL). After 15 min, blood samples were collected in tubes containing EDTA and aprotinin. The samples were immediately centrifuged at 4°C at 3,000 rpm for 15 min. Plasma concentrations of OXT were measured as described in the measurement of plasma OXT section.

### 5.8 Sub‐Chronic Nasal Treatment of OXT

Forty‐seven to 50 weeks old WT male mice were habituated for 10 days in individual cages. 10 μg/10 μL of OXT (4084‐v; Peptide Institute, Osaka, Japan) or vehicle (0.9% saline) was dropped into the nasal cavity for 10 consecutive days. Body weight and food intake were measured for 10 days. Ten days of OXT treatment was enough term to reduce body weight via changing energy metabolism (Maejima et al. 2017, 2011). For qRT‐PCR and western blotting, on Day 11 after the beginning of nasal OXT treatment, mice were intraperitoneally injected with a mixture of three types of anesthetic agents (10 mL/kg), and collected plasma samples, hypothalamic (0.14 mm to −2.06 mm from bregma), and hippocampal (−1.22 mm to −2.54 mm from bregma) tissues were collected. For immunostaining, mice were perfused intracardially with 4% PFA and 0.2% picric acid.

### 5.9 Western Blot Analysis

Applicable animals were anesthetized with a mixture of three types of anesthetic agents. Their brains were removed and hypothalamus (0.14 mm to −2.06 mm from bregma) and hippocampus (−1.22 mm to −2.54 mm from bregma) tissues were dissected under a microscope. The tissue samples were lysed in radioimmunoprecipitation assay buffer containing 50 mM Tris–HCl (pH 8.0), 150 mM NaCl, 1% Nonidet P‐40, 0.5% sodium deoxycholate, and 0.1% SDS with 1% protease inhibitor cocktail. This homogenate lysate was centrifuged at 4°C for 10 min at 12,000 g. The supernatants were collected, and protein concentrations were measured. The samples were dissolved in 2× SDS sample buffer and boiled at 95°C for 5 min. A quantity of 20 μg total protein from the samples was loaded onto a 10% or 15% polyacrylamide gel (C‐PAGEL HR 15 L or 10 L, ATTO, Tokyo, Japan). The samples were separated and the proteins were transferred from the gel onto PVDF membranes at 85 mA for 60 min with a transfer buffer (AE‐1465, ATTO, Tokyo, Japan). The membrane was washed several times with PBS containing 0.05% Tween 20 (PBST) and blocked with 5% skim milk (190–12,865, Wako, Osaka, Japan) in PBST for 60 min at room temperature. The membrane was then incubated with rabbit polyclonal anti‐COX IV antibody (1:1000; PM063, MBL, Tokyo, Japan) or anti‐TET2 rabbit polyclonal antibody (1:1000; 21,207‐1‐AP, proteintech, IL) rabbit monoclonal and rabbit polyclonal anti‐GAPDH antibody (1:5000; GTX100118, GeneTex, CA) overnight at 4°C. The membrane was washed with PBST and incubated with HRP‐conjugated secondary antibodies against rabbit IgG (1:1000; PI‐1000, VECTOR LABORATORIES, CA) for 60 min at room temperature, washed, and immunoreactive bands were detected using the ELC Prime Western Blotting Detection Reagent (GE Healthcare, Uppsala, Sweden).

### 5.10 Primary Neuronal Culture

Forebrain neurons from embryonic Day 14.5 (E14.5) C57BL/6J mice or OXTR null mice were prepared based on a previous article with slight modification (Numakawa et al. 2002). In brief, forebrain neurons were dissociated by papain (25 unit/mL; P4726, Merk, Darmstadt, Germany) in PBS (−) containing 0.01% DNAse I (047‐26,771; FUJIFILM, Tokyo, Japan), 0.02% L‐systein (FUJIFILM, Tokyo, Japan), 0.02% bovine serum albumin, and 0.5% glucose for 15 min at 37°C. After incubation, cells were centrifuged at 230 g for 2 min. The supernatant was removed, and D‐MEM/Ham's F‐12 (048‐29,785, FUJIFILM, Tokyo, Japan) containing 5% fetal bovine serum, 5% horse serum, and 1% penicillin–streptomycin was added. Following gentle trituration, the medium containing separated neurons was passed through the 70 μm mesh. The medium containing neurons was centrifuged at 230 g for 2 min. The supernatant was removed, and isolated cells were seeded (1 × 10^6^ cells/ml) onto 35‐mm culture or glass bottom dishes coated with polyethyleneimine (Wako, Osaka, Japan). Twenty‐four hours after seeding, cytosine arabinoside (AraC; 5 μM; Sigma‐Aldrich, MO) was added to the medium in order to inhibit glial cell proliferation. Half of the medium was replaced with fresh medium every 3 days.

To examine the neuronal aging, senescence‐associated β‐galactosidase (SA‐β‐gal) was stained by using a cellular senescence kit (GXS0003, OZ Bioscience, Marseille, France) at 7, 21, and 35 days after seeding. After staining SA‐β‐gal, plates were fixed by 10% formalin and stained with hematoxylin to detect nuclei. Images from 10 areas were acquired from three plates, and the number of SA‐β‐gal‐positive neurons and nuclei was counted.

### 5.11 Immunocytochemistry for COX IV

Primary neurons from WT mice after seeding 14 and 35 days, or primary neurons from OXTR null mice after seeding 35 days, were fixed by 4% PFA and 0.2% picric acid for 60 min. After washing the dishes, 0.1% Triton‐X PBS was added to the dishes for 30 min. In order to block autofluorescence, True Black (#23007, Biotium, CA, USA) was treated for 5 min. After washing, the dishes were incubated with PBS containing 2% BSA and 5% NGS (blocking solution) for 60 min. After blocking, the dishes were incubated with blocking solution containing anti‐COX IV rabbit polyclonal antibody (1:500; PM063, MBL, Tokyo, Japan) overnight at 4°C. The next day, the dishes were washed with PBS and incubated with blocking solution containing Alexa fluor 488‐labeled goat antirabbit IgG (1:400; Life Technologies, CA). The images of five microscopic fields were acquired from five to six plates in each group. The intensity of COX IV fluorescence and the number of DAPI‐positive cells were analyzed by NIH image software (Image J, National Institute of Health).

### 5.12 Ca^2+^ Imaging in Primary Neurons

Primary neurons obtained from WT and OXTR null mice at Day 30–35 were loaded with Fura‐2/AM (1 μM, Dojindo, Kumamoto, Japan) for 30 min in a CO_2_ incubator, according to our previous report (Maejima et al. 2021). In brief, the dishes were placed in a chamber for Ca^2+^ imaging and superfused with Krebs Ringer Bicarbonate buffer (KRB) at 1 mL/min at 30°C. Cells loaded with fluorescent dye were illuminated by alternating between 340 and 380 nm, and the resultant fluorescent images were captured. The ratio (F340/F380) images were produced by Aquacosmos version 2.6 (Hamamatsu Photonics, Hamamatsu, Japan). Amplitudes of [Ca^2+^]_i_ responses to OXT (10^−7^ M) were calculated by subtracting the prestimulatory basal [Ca^2+^]_i_ ratio (average of 3 min: control) from the peak [Ca^2+^]_i_ ratio during application of agents. In the last measurement, 30 mM KCl was applied to check cell viabilities.

### 5.13 The Treatment of OXT and OXTR Antagonist (H4928) for Primary Neurons

Primary neurons obtained from WT and OXTR null mice at Day 35 were treated with OXTR antagonist (10^−7^ M, [d(CH2)5 1, Tyr (Me)2, Orn8]‐Oxt, H4928, Bachem, Budendorf, Switzerland) or OXT (10^−9^ M) for 7 days. Medium containing each agent was replaced with fresh medium every 2 days. On Day 7 after the beginning of treatment, the dishes were washed with PBS, and western blotting was performed for COX IV. The methods are the same as those described in the section of western blot analysis.

### 5.14 Statistical Analysis

All data are presented as mean ± SEM. The comparison of data from two groups was performed using Student's *t*‐test. The comparison of data from multiple groups was performed using one‐way ANOVA followed by Tukey's multiple range test. GraphPad Prism 9.0 (GraphPad Software, CA) was used for these statistical analyses. All statistical tests were two‐tailed, with values of 0.05 considered statistically significant.

## Author Contributions

Conceptualization, Project administration: Y.M. Methodology: Y.M., S.H., K.N., S.T., T.O. Investigation: Y.M., S.Y., M.Y., S.T. Visualization: Y.M., S.T. Funding acquisition, writing – original draft: Y.M., K.S. Supervision: K.S., H.W. Writing – review and editing: Y.M., S.Y., M.Y., S.H., T.O., S.T., K.N., H.W., K.S.

## Conflicts of Interest

The authors declare no conflicts of interest.

## Supporting information


**Data S1:** acel70198‐sup‐0001‐Supinfo1.pdf.

**Data S2:** acel70198‐sup‐0002‐Supinfo2.docx.

## Data Availability

The data that support the findings of this study are available from the corresponding author upon reasonable request.
