# Supplementary material for: Oxytocin Enhances Demethylation Through TET Enzyme Expression in Neurons of Aged Mice: Oxytocin as a Potential Antiaging Peptide
Source: Aging Cell. 2025 Aug 11;24(10):e70198. doi: 10.1111/acel.70198 (PMC12507420; doi:10.1111/acel.70198)
Supplement: Supplementary file 1 — Data S1: acel70198‐sup‐0001‐Supinfo1.pdf. [file ACEL-24-e70198-s002.pdf]

AGING CELL AUTHOR CHECKLIST. *Authors should submit this checklist together with their manuscript. Please ensure that you have read the Author Guidelines in detail before submission.*

Title	Oxytocin enhances demethylation through TET enzyme expression in neurons of aged mice: oxytocin as a potential anti-aging peptide						
Authors	Yuko Maejima, Shoko Yokota, Megumi Yamachi, Shizu Hidema, Tomoyuki Ono, Shu Taira, Katsuhiko Nishimori, Heidi de Wet, Kenju Shimomura						
Manuscript Type	Research article						
Total Character Count (including spaces)[1]	48,362 characters						
Word count of Summary[2]	241 words						
Number of papers cited in the References[3]	66 references						
Listing of all Tables (Table1, Table 2 etc)[4]	Table 1						
Figure specifications (please complete one row per figure)[5] *Figure no.*	Colour *(yes/no)*	Greyscale *(yes/no)*	Black and white *(yes/no)*	Single column (80mm) *(yes/no)*	Double column (180mm) *(yes/no)*	Size of figure at full scale (mm x mm) *(insert details)*	Smallest font size used in the figure at full scale (minimum 6pt) *(insert details)*
Figure 1	Yes	no	no	no	no	263 × 156	11
Figure 2	Yes	no	no	no	no	179 × 342	11
Figure 3	Yes	no	no	no	no	249 × 190	8
Figure 4	Yes	no	no	no	no	273 × 203	8
Figure 5	Yes	no	no	no	no	264 × 186	8
Figure 6	Yes	no	no	no	no	193 × 286	6

[1] The maximum character count allowed is 50,000 (incl. spaces) for Primary Research Papers and Reviews, 10,000 for Short Takes.

[2] Summary should not exceed 250 words.

[3] Primary Research Papers can contain a maximum of two tables. If more are needed they should replace some of the Figures or can be placed in the Supporting Information.

[4] A maximum of 45 references is allowed for Primary Research Papers and 20 references for Short Takes.

[5] A Primary Research Paper may contain up to 6 figures and a Short Take up to 2 figures. Authors are encouraged to provide figures in the size they are to appear in the journal and at the specifications given.
