# Supplementary material for: Oxytocin Enhances Demethylation Through TET Enzyme Expression in Neurons of Aged Mice: Oxytocin as a Potential Antiaging Peptide
Source: Aging Cell. 2025 Aug 11;24(10):e70198. doi: 10.1111/acel.70198 (PMC12507420; doi:10.1111/acel.70198)
Supplement: Supplementary file 2 — Data S2: acel70198‐sup‐0002‐Supinfo2.docx. [file ACEL-24-e70198-s001.docx]

*Supplemental information*

**Oxytocin enhances demethylation through TET enzyme expression in neurons of aged mice: oxytocin as a potential anti-aging peptide**

Yuko Maejima^1,2,3^, Shoko Yokota^1^, Megumi Yamachi^1^, Shizu Hidema^1^, Shu Taira^4^, Katsuhiko Nishimori^2^, Heidi de Wet^3^, Kenju Shimomura^1,2^

*^1^Department of Bioregulation and Pharmacological Medicine, Fukushima Medical University School of Medicine, Fukushima-shi, 960-1295, Fukushima, Japan*

*^2^Departments of Obesity and Inflammation Research, Fukushima Medical University School of Medicine, Fukushima-shi, 960-1295, Fukushima, Japan*

*^3^Department of Physiology, Anatomy and Genetics, Sherrington Building, University of Oxford, United Kingdom*

*^4^* *Faculty of Food and Agricultural Sciences, Fukushima University, Fukushima-shi, 960-1248, Fukushima, Japan*


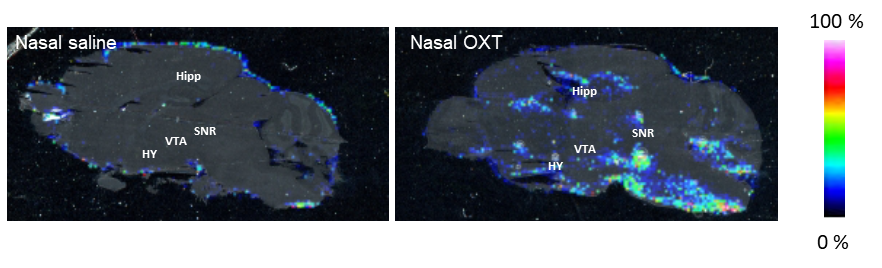
**Supplemental Fig.1 Mass spectrometry image of OXT in the sagittal section from aged mouse treated with saline (left panel) or OXT (right panel).**

HY: hypothalamus, Hipp: hippocampus, VTA: ventral tegmental area, SNR: substantia nigra.

After 30 min of nasal saline or OXT treatment (10 μg/10 μl) to aged WT mice (45 weeks old), mice were anesthetized with a mixture of three types of anesthetic agents, and brains were removed. These brains were embedded into a super cryo-embedding medium (Section Lab Co., Ltd., Hiroshima, Japan), flash-frozen in liquid N2 and stored at −80°C until use.

The left hemisphere was embedded in cryo-embedding medium (Section Lab Co., Ltd., Hiroshima, Japan) and cut into serial sagittal sections (8 μm) using a cryostat (NX70, PHC, Tokyo, Japan).

The brain sections were sprayed with matrix (CHCA-acetonitrile/water/trifluoroacetic acid = 70/49.9/0.1) using an automated pneumatic sprayer (TM-Sprayer, HTX Tech., Chapel Hill, NC). Ten passes were sprayed with the following conditions; flow rate 120 μl/min, air flow 10 psi, and nozzle speed 1100 mm/min. In order to detect the laser spot locations, the sections were scanned, and laser spot areas (200 shots) were detected with a spot-to-spot center distance (100 μm) in each direction of the brain. Signals between *m/z* 500 and 1500 were adjusted. The section surface was irradiated with YAG laser pulses in the positive ion detection mode. The laser power was optimized to minimized in-source decay of targets. Obtained MS spectra were reconstructed to an image with a mass bin width of *m/z* ± 1.0 from the precise mass using FlexImaging 4.0 software (Bruker Daltonik GmbH). The peak intensity value of the spectra was normalized by dividing it with the total ion current (TIC) to achieve semi-quantitative comparisons between OXT treatment and control mice. An optical image of brain section was produced using a scanner (GT-X830: Epson, Japan), followed by MALDI-TOF imaging MS of the section.　The relative total ion counts for OXT in the entire brain stem, HY and VTA revealed ratios of 1: 21, 1:1.1and 1:15.5 for mice control and treatment with OXT, respectively.

**
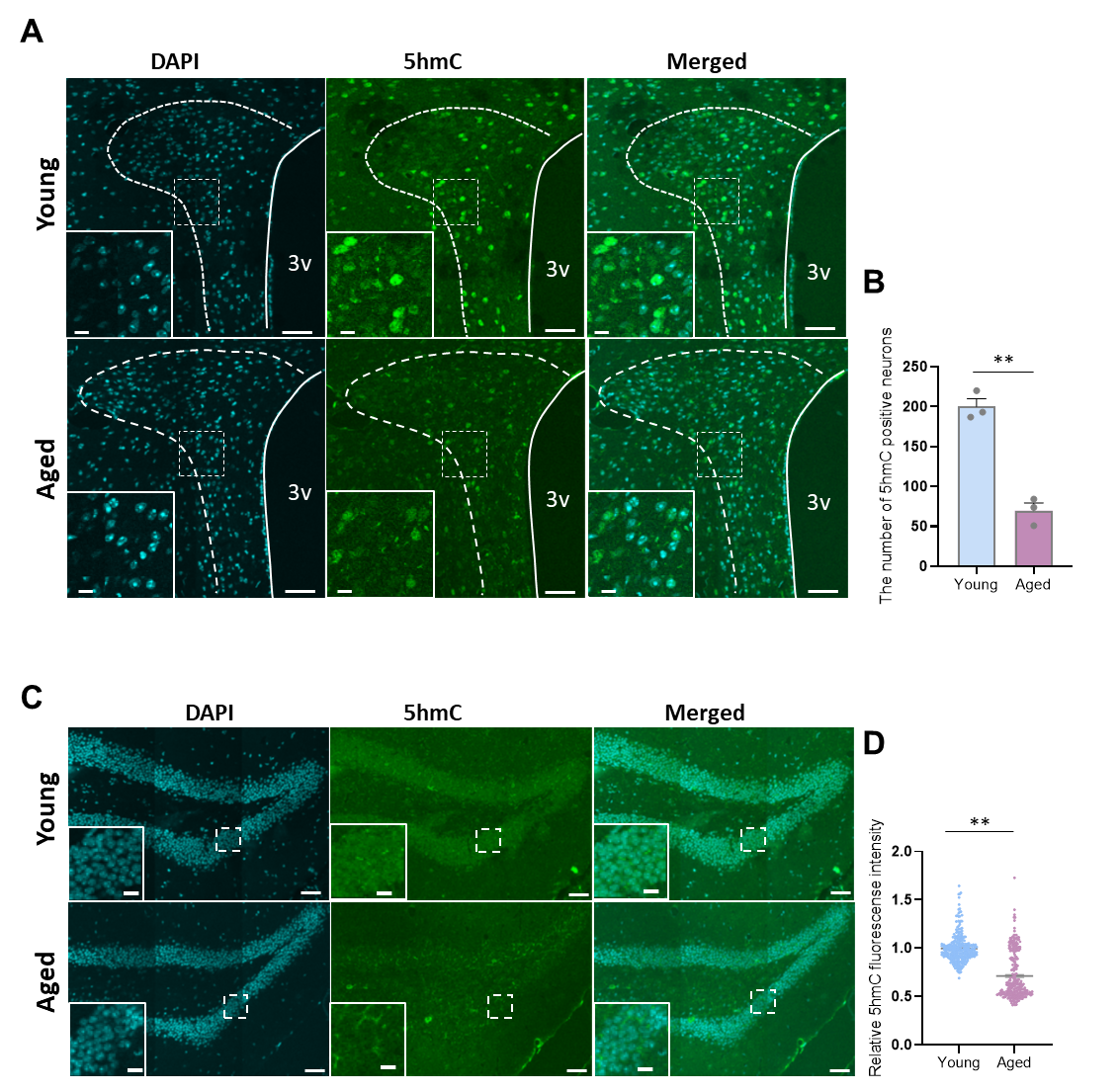
Supplemental Fig. 2 Comparing 5hmC protein expression in young and aged mice.**

Young (9 weeks) and aged (47 weeks) were intraperitoneal injected with a mixture of three types of anesthetic agents (10 ml/kg) and perfused intracardially with 4% paraformaldehyde (PFA) and 0.2% picric acid. Serial coronal sections (40 μm) were collected from each mouse using a freezing microtome. The sections were washed in PBS and incubated with 0.1% Triton-X for 30 min. For antigen retrieval, sections were incubated with 4N HCl for 15 min. After rinse with distilled water, the sections were incubated Tris-HCl buffer (0.1M, pH 8.5) for 10 min. In order to block autofluorescence, True Black (#23007, Biotium, CA. USA) was treated for 5 min. The sections were washed in PBS and incubated for 1 hr in a blocking solution comprising of 2% bovine serum albumin (BSA), and 5% normal goat serum (NGS). Sections were incubated with anti-5hmC mouse monoclonal antibody (1:500; 51660, Cell Signaling, MA) in blocking solution over night at 4°C. Then sections were incubated with Alexa flour 488-labelled goat anti-mouse IgG (1:400; Life Technologies, CA) for 40 min. Sections were mounted on glass slides and covered. Confocal fluorescence images of PVN and dentate gyrus of hippocampus were acquired by FV10i (Olympus, Tokyo, Japan). 5hmC positive cells in PVN were counted per section and averaged. The intensity of 5hmC in each DAPI positive area of dentate gyrus were analyzed by image J (National Institute of Health).

**A:** Representative image of 5hmC staining in young (upper panels) and aged (bottom panels) mice of PVN. Scale = 50 μm. The images located in left bottom are enlarged images in dotted area. Scale = 10 μm. **B:** The number of 5hmC positive neurons per section. ***p* < 0.01, unpaired *t*-test. *n* = 3. **C:** Representative image of 5hmC staining in young (upper panels) and aged (bottom panels) mice of dentate gyrus of hippocampus. Scale = 50 μm. The images located in left bottom are enlarged images in dotted area. Scale = 10 μm. **D:** Relative 5hmC fluorescence intensity in nuclear DAPI area. The relative fluorescence intensity of 5hmC in young mice was corrected to 1. ***p* < 0.01, unpaired *t*-test. *n* = 306, 256 (from 3 mice).

**
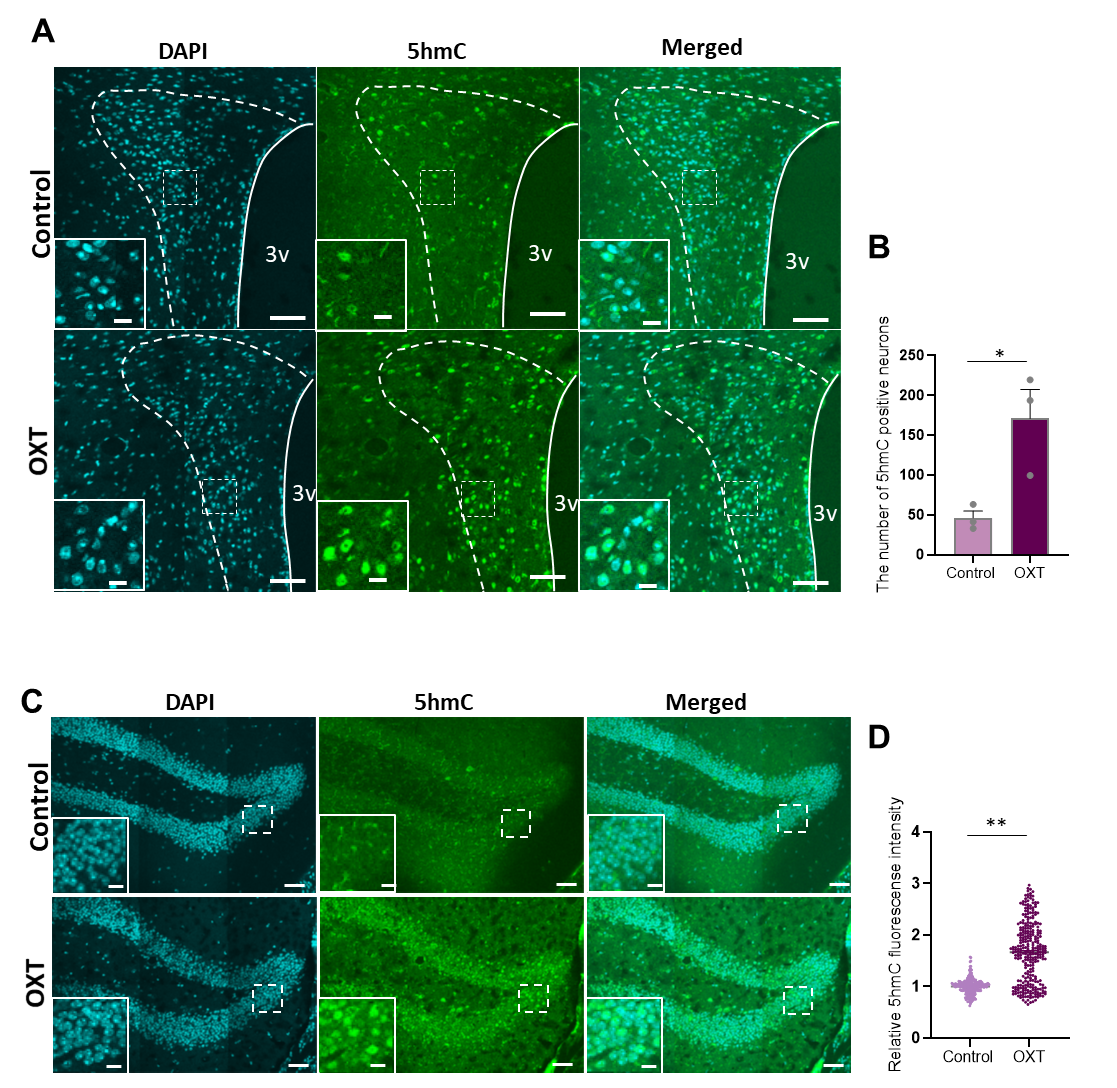
Supplemental Fig. 3 5hmC protein expression after nasal OXT treatment in aged mice.**

Animals were nasally treated saline (47-75 weeks) or 10 μg OXT (47-75 weeks) for 10 days. Perfusion, 5hmC staining and analyze were performed same with supplemental Fig.1.

**A:** Representative image of 5hmC staining in control saline (upper panels) and OXT treated (bottom panels) mice of PVN. Scale = 50 μm. The images located in left bottom are enlarged images in dotted area. Scale = 10 μm. **B:** The number of 5hmC positive neurons per section. **p* < 0.05, unpaired *t*-test. *n* = 3. **C:** Representative image of 5hmC staining in control (upper panels) and OXT (bottom panels) treated mice of dentate gyrus of hippocampus. Scale = 50 μm. The images located in left bottom are enlarged images in dotted area. Scale = 10 μm. **D:** Relative 5hmC fluorescence intensity in nuclear DAPI area. The relative fluorescence intensity of 5hmC in control mice was corrected to 1. ***p* < 0.01, unpaired *t*-test. *n* = 314, 297 (from 3 mice).

**
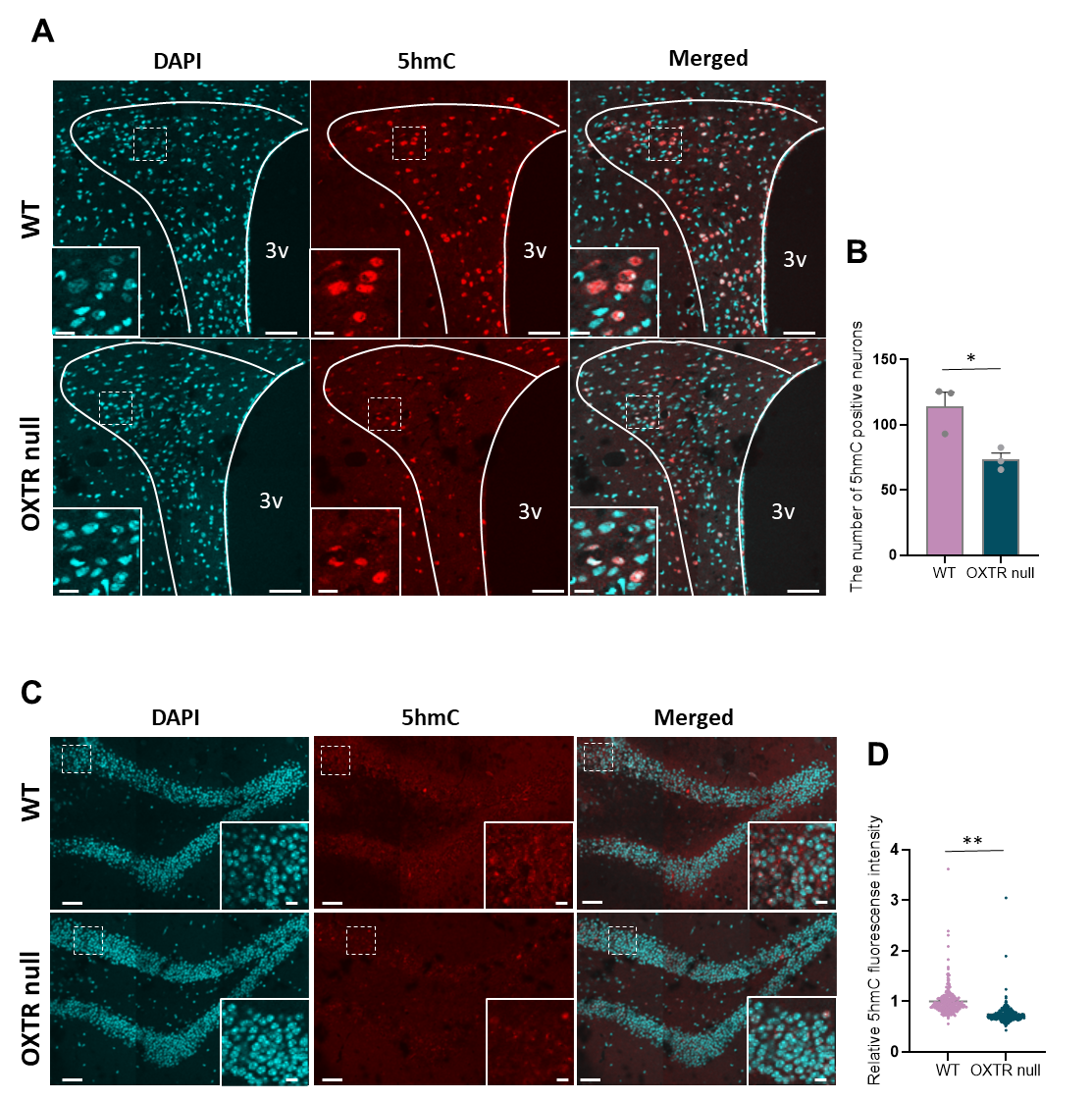
Supplemental Fig. 4 Comparing 5hmC protein expression in aged wild and OXTR null mice.**

84 weeks WT and OXTR null mice were used in this experiment. Perfusion, 5hmC staining and analyze were performed same with supplemental Fig.1.

**A:** Representative image of 5hmC staining in WT (upper panels) and OXTR null (bottom panels) mice of PVN. Scale = 50 μm. The images located in left bottom are enlarged images in dotted area. Scale = 10 μm. **B:** The number of 5hmC positive neurons per section. **p* < 0.05, unpaired *t*-test. *n* = 3. **C:** Representative image of 5hmC staining in WT (upper panels) and OXTR null (bottom panels) mice of dentate gyrus of hippocampus. Scale = 50 μm. The images located in right bottom are enlarged images in dotted area. Scale = 10 μm. **D:** Relative 5hmC fluorescence intensity in nuclear DAPI area. The relative fluorescence intensity of 5hmC in WT mice was corrected to 1. ***p* < 0.01, unpaired *t*-test. *n* = 280, 251 (from 3 mice).


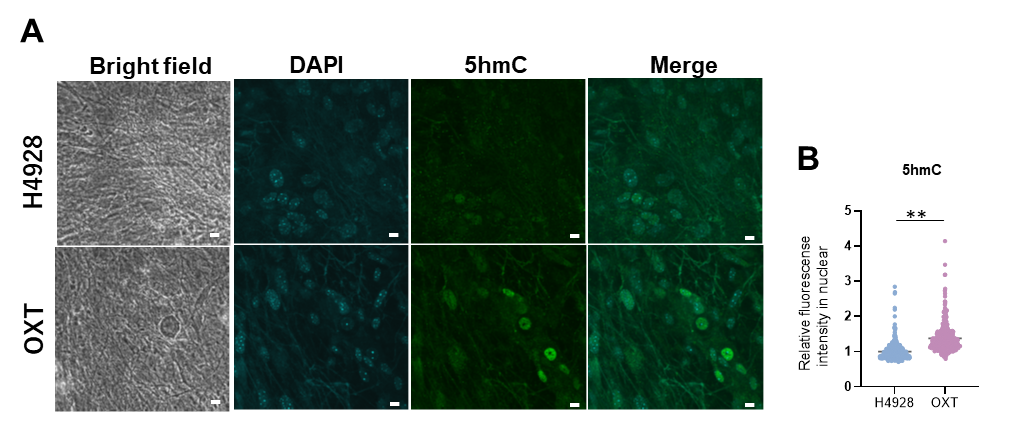


**Supplemental Fig. 5 Comparing 5hmC protein expression in cultured primary neurons.**

Primary neurons obtained from WT mice at day 35 were treated with OXTR antagonist (10^-7^ M, [d(CH2)5 1, Tyr (Me)2, Orn8]-Oxt, H4928, Bachem, Budendorf, Switzerland) or OXT (10^-9^ M) for 7 days. 5hmC staining was performed same with supplemental Fig.1. The intensity of 5hmC in each DAPI positive area were analyzed by image J (National Institute of Health).

**A:** Representative image of 5hmC staining in aged primary neurons treated with H4928 (upper panels) and OXT (bottom panels). Scale = 10 μm. **B:** Relative 5hmC fluorescence intensity in nuclear DAPI area. The relative fluorescence intensity of 5hmC in WT mice was corrected to 1. ***p* < 0.01, unpaired *t*-test. *n* = 406, 409 (from 4 plates).

**
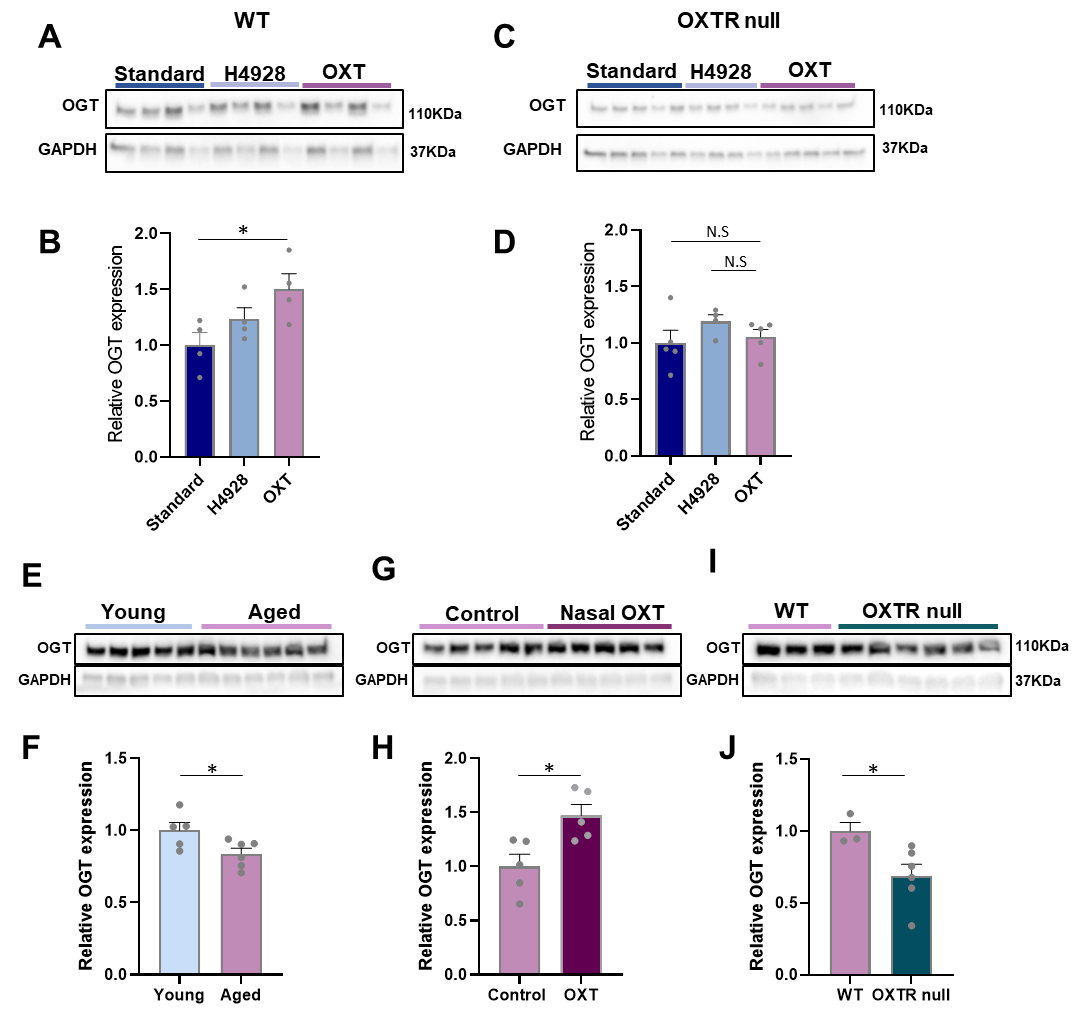
**

**Supplemental Fig. 6 The impact of OXT/OXTR on O-GlcNAc transferase (OGT)**

**A;** The image of western blotting of OGT in primary neurons from WT, treated with H4928 (10^-7^ M), OXT (10^-9^ M) or non-treated (standard medium). **B;** The relative expression of OGT to GAPDH in primary neurons from WT, treated with H4928, OXT or non-treated. OGT expressions in non-treated neurons are correct to 1. * *p* < 0.05, one-way ANOVA followed by Tukey’s multiple range test, *n* = 4 (plate). **C;** The image of western blotting of OGT in primary neurons from OXTR null mice, treated with H4928 (10^-7^ M), OXT (10^-9^ M) or non-treated standard medium. **D;** The relative expression of OGT to GAPDH in primary neurons from OXTR null mice, treated with H4928, OXT or non-treated. OGT expressions in non-treated neurons are correct to 1. N.S; no significant differences, one-way ANOVA followed by Tukey’s multiple range test, *n* = 4-5 (plate). **E;** The image of western blotting of OGT in hippocampus from young (9 weeks) and aged (93 weeks) mice. **F;** The relative expression of OGT to GAPDH in young and aged hippocampus. OGT expressions in non-treated neurons are correct to 1. * *p* < 0.05, one-way ANOVA followed by Tukey’s multiple range test, *n* = 5-6. **G;** The image of western blotting of OGT in hippocampus from aged mice (62 weeks) treated with saline (control) or OXT (10 μg/μl/day) for 10 days. **H;** The relative expression of OGT to GAPDH in hippocampus from aged mice. OGT expressions in non-treated neurons are correct to 1. * *p* < 0.05, one-way ANOVA followed by Tukey’s multiple range test, *n* = 5. **I;** The image of western blotting of OGT in hippocampus from aged WT (80-85 weeks) and OXTR null (81-83 weeks) mice. J: The relative expression of OGT to GAPDH in hippocampus from aged WT and OXTR null mice. OGT expressions in non-treated neurons are correct to 1. * *p* < 0.05, one-way ANOVA followed by Tukey’s multiple range test, *n* =3-6.

**
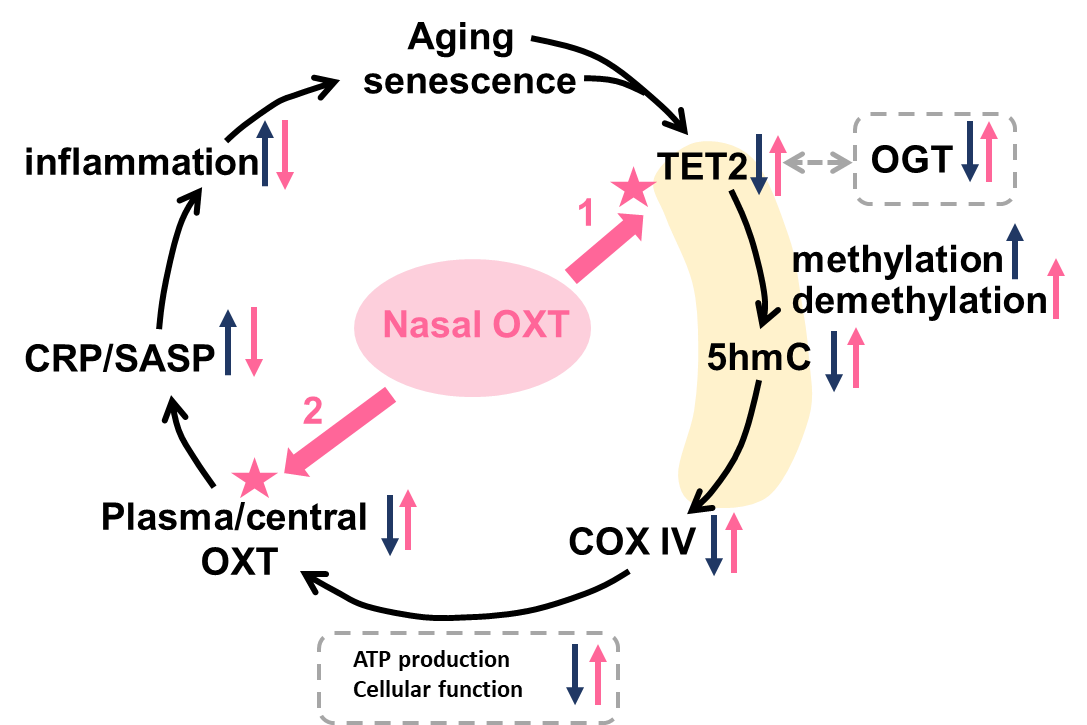
Supplemental Fig. 7 The role of OXT in vicious cycle of senescence**

DNA methylation is increased with age as a consequence of decreasing TET2, O-GlcNAc transferase (OGT) and 5hmC expression (Gontier et al. 2018 Wheatley et al. 2019,) and these changes are linked to decreased COX IV expression and function(Liu et al., 2020) . Decreased COX IV expression and activity will in turn impact on the electron transport chain, resulting in decreased ATP production and compromised mitochondrial and cellular function (Li Y et al., 2006)

It is postulated that increased methylation and decreased cellular function induce a decline in central OXT expression and plasma OXT levels, which induce systemic inflammation with increasing senescence-associated secretory phenotype (SASP) and CRP. SASP, including inflammatory cytokines, accelerate senescence. In this vicious cycle of senescence, we propose that there are two intervention points for OXT. The first result of OXT nasal treatment is a OXTR mediated increased expression of both TET2 and OGT which would promote DNA demethylation and the subsequent recovery of COX IV expression, cellular function, central OXT expression, and plasma OXT levels. Consequently, inflammation is suppressed via decreased SASP secretion. The second intervention point is that increased peripheral OXT levels by nasal administration itself can decrease SASP directly, with a resultant decrease in inflammation and anti-ageing effects.

Blue arrows indicate the effect of aging/senescence. Pink arrows indicate the effect of OXT. Star (★) marks indicate the action points for OXT. Area which need further investigation are circled by a gray dotted line.
